# Better insurance could effectively mitigate the increase in economic growth losses from U.S. hurricanes under global warming

**DOI:** 10.1126/sciadv.add6616

**Published:** 2023-01-04

**Authors:** Christian Otto, Kilian Kuhla, Tobias Geiger, Jacob Schewe, Katja Frieler

**Affiliations:** ^1^ Potsdam Institute for Climate Impact Research, Telegrafenberg A56, Potsdam, Germany.; ^2^ Deutscher Wetterdienst, Klima und Umwelt, Potsdam, Germany.

## Abstract

Global warming is likely to increase the proportion of intense hurricanes in the North Atlantic. Here, we analyze how this may affect economic growth. To this end, we introduce an event-based macroeconomic growth model that temporally resolves how growth depends on the heterogeneity of hurricane shocks. For the United States, we find that economic growth losses scale superlinearly with shock heterogeneity. We explain this by a disproportional increase of indirect losses with the magnitude of direct damage, which can lead to an incomplete recovery of the economy between consecutive intense landfall events. On the basis of two different methods to estimate the future frequency increase of intense hurricanes, we project annual growth losses to increase between 10 and 146% in a 2°C world compared to the period 1980–2014. Our modeling suggests that higher insurance coverage can compensate for this climate change–induced increase in growth losses.

## Teaser

Better insurance could help compensate for rising economic growth losses from US hurricanes under global warming.

## INTRODUCTION

Already in the present climate, hurricanes in the North Atlantic cause substantial economic losses in the United States. Between 1980 and 2014, these storms caused more than US$ 410 billion in direct economic losses with annual losses peaking at more than US$ 100 billion in 2005 according to Munich Re’s NatCatSERVICE database (*1*). Moreover, there is increasing empirical evidence that, in addition to these direct losses, tropical storms can substantially reduce economic growth of affected countries for more than a decade (*2*–*4*). These long-term growth impacts may have important implications for the adaptation to, and coping with, the impacts of tropical storms under global warming, because there is strong evidence that the proportion of intense storms of the two highest categories 4 and 5 on the Saffir-Simpsons scale may increase (*5*–*8*). There are at least two mechanisms through which this increase could overcompensate a possible mild decline of the overall number of tropical storms (*5*, *6*) driving up economic losses. First, the most intense storms cause disproportionately larger direct economic losses than smaller storms. For instance, major category 4 and 5 hurricanes have accounted for almost half of normalized economic damage from all hurricanes that made landfall in the United States in the period 1900–2005 despite representing only about 6% of landfall events (*9*). Second, if the increase in the proportion of intense storms overcompensates the possible decline in overall storm number, then intense storms become more frequent. This would leave, on average, less time for the economy to recover in between consecutive intense storms; incomplete recovery has been identified as one main factor that may increase the vulnerabilities of the economy to climate extremes and thereby drive up losses (*10*, *11*). Moreover, the activity of hurricanes in the North Atlantic—as of other main categories of extreme weather events such as floods and droughts (*12*)—is influenced by (multi-)decadal modes of climate variability such as the Atlantic Multidecadal Oscillation (*13*). There is an ongoing discussion in the literature whether this has resulted in a statistically significant clustering of U.S. hurricanes in the historical period (*13*–*15*). However, under global warming, the clustering of hurricanes might intensify (*12*). This would render incomplete recoveries more likely and increase hurricane damage.

Catastrophe insurance is discussed as a means to reduce vulnerabilities of the economy to extreme weather events by shortening the recovery time in the disaster aftermath (*16*–*19*), and it may thereby even promote economic growth on the macroeconomic level (*20*–*22*). These promising findings may explain the rising popularity of multilateral climate risk insurance schemes and the G20 InsuResilience Global Partnership initiative (*23*). However, it remains an open question whether better insurance will be sufficient to counteract climate change impacts in a warming world (*24*, *25*).

Progress in answering this question has been also made difficult by the limitations of state-of-the-art climate integrated assessment models (IAMs). These standard workhorses for climate policy assessments [see (*26*) for detailed review on IAMs)]—such as the seminal Dynamic Integrated Climate-Economy (DICE) model (*27*), which is used by the U.S. government to estimate the cost of carbon emissions to society—have been criticized for not being able to appropriately account for the impacts of climate extremes (*28*, *29*).

The main reason is that the coarse temporal spatial resolution of most models [typically 1 to 10 years and about 10 world regions where climate impacts are scaled in terms of global mean temperature (GMT) changes] simply does not allow for the representation of individual extreme weather events; potentially important nonlinearities arising from a disproportional increase of total economic losses with impact intensity or from incomplete recovery between consecutive events cannot be resolved. In consequence, IAM-based studies usually report relatively small, or even negligible, impacts of climate extremes on the economy (*30*) and cannot reproduce the (mostly adverse) long-term impacts of these events on economic growth reported in the recent economic literature (*2*, *3*, *31*). Further, most state-of-the-art IAMs are deterministic. In such a setup, the economic agents can account for projected changes in (mean) climate damages with global warming but not for the associated uncertainties in climate impact projections due to fluctuations induced by extreme weather events or our missing knowledge about irreversible regime changes (*32*) when climate tipping points are transgressed (*33*, *34*). This can lead to a substantial underestimation of the risks climate change poses for societies (*35*) and economies (*36*) and, in consequence, a lack in climate ambition (*37*). Over the last years, new modeling approaches such as stochastic IAMs (*38*) and agent-based IAMs (*39*) have emerged that aim for a better representation of climate risks. In stochastic IAMs, representative agents account for the risk of extreme climate change impacts in their expectation formation. In agent-based IAMs, additionally, the assumption of representative agents is relaxed, and the macroeconomic dynamics emerges from the interaction of multiple, heterogeneous economic agents. This allows accounting for differences in the perception of, and response to, climate risks. Both modeling approaches substantially drive up the expected cost of greenhouse gas emissions for societies, the social cost of carbon (*36*–*39*).

Here, we particularly focus on a better representation of the long-term impacts of extreme weather events on economic growth. To this end, we build a simple—and transparent—event-based neoclassical growth model for a national economy (cf. Eqs. 1A to 1C). The model accounts for losses to the stock of physical assets (shocks) that result from individual landfall events. We assume that these shocks are nonmarginal in the sense that they affect all “productivity layers” of assets equally instead of merely destroying the least efficient assets (*40*, *41*). In consequence, output is reduced by the same factor as the stock of physical assets, and, in consequence, output losses are larger than for marginal shocks. In line with empirical evidence for intense reconstruction activities in the (immediate) disaster aftermath (*42*), we assume that the reconstruction of destroyed capital provides higher marginal returns than the growth of the stock of productive assets due to investments into new technologies (cf. Eqs. 6A and 6B). In consequence, the recovery of the economy can be divided into a first phase of rapid reconstruction of destroyed capital and a second phase, where the economy slowly approaches the balanced growth path (BGP) of the unperturbed system. Further, reconstruction investments can be capped in the disaster aftermath to describe inefficiencies slowing down the economic recovery such as scarcity of trained labor and building materials and other financial and technical constraints in the reconstruction process (cf. Eq. 7) (*40*), which slows down the recovery speed in the first phase. Further, we integrate a compulsory nonprofit hurricane insurance financed by a flat fee on all citizens, regardless of their individual risk (*16*). This insurance scheme represents a precautionary savings mechanism where premiums accumulated in normal times are issued to affected households in the disaster aftermath. Because there are several empirical studies reporting that insurance payouts facilitate and speed up the reconstruction process in the disaster aftermath [cf. (*16*) for a recent review] (*43*–*45*), we assume that the investment cap can be temporarily exceeded by the amount of insurance payouts (cf. Eq. 7).

The discussed insurance scheme resembles tax financed and nonprofit public insurance schemes already implemented in the United States today such as U.S. National Flood Insurance Program (NFIP) managed by the Federal Emergency Management Agency (*46*), the Florida Hurricane Catastrophe Fund, or the California Earthquake Authority [see (*47*) for a detailed review on catastrophe insurance programs in Organization for Economic Co-operation and Development countries]. However, because here we are interested in finding the upper limit for the share of climate change–induced losses that insurance can compensate for, the discussed insurance scheme is in two aspects more comprehensive than these existing insurance schemes: First, by assuming that all assets are insurable against hurricane risks, we exclude potential issues regarding the uninsurability of losses, e.g., particularly disaster-prone locations. Second, by considering insurance to be compulsory, we exclude issues of limited insurance uptake by the population.

In the standard calibration of the model, the insurance coverage is set to 50% the average ratio of insured losses in the United States between 1980 and 2014 according to the NatcatSERVICE database (*1*), and reconstruction investments are capped to 0.2% of weekly output following (*40*). With this model calibration and driving the model with the (direct) asset losses of the 88 historical hurricanes that made landfall in the United States in the period 1980–2014 according to the NatCatSERVICE database (*1*), we obtain average annual output growth losses of about 0.024 percentage points (p.p.), which fall well into the range constrained by the recent climate econometrics literature [e.g., Bakkensen and Barrage (*48*) and Hsiang and Jina (*4*) report average annual growth losses of about 0.006 and 0.880 p.p., respectively]. Throughout the manuscript, we will use the word “hurricanes” also for tropical cyclones of tropical storm strength and define a hurricane to make landfall if a portion of its wind field touches land with at least tropical storm strength, thereby accounting for hurricane damages even if the hurricane’s core did not touch land (*49*).

The asset losses caused by the hurricanes that made landfall in the historical period are very unequally distributed; while the asset losses of the most destructive storms exceeded 0.1% of the real national gross domestic product (GDP) in the year of landfall, the majority of the storms caused asset losses of less than 0.001% of GDP (fig. S3). We first study how this heterogeneity in asset losses has affected economic growth in the period 1980–2014. We then project increases in growth losses that would arise from changes in the proportion of intense storms, overall storm number, and associated changes in impact heterogeneity in a Paris-compatible 2°C world and in a world that is 2.7°C warmer than in preindustrial times corresponding to the median warming estimate by 2100 under the currently implemented or enacted policies (“current policy path”) (*50*). Because there is substantial uncertainty on how hurricane climatology will change with global warming, and the magnitude of the effect strongly depends on the underlying methodology used to estimate this change (*6*), we consider two different approaches at both ends of the uncertainty range. In addition, we assess the efficacy and limits of disaster insurance in mitigating the climate change–induced increase in growth losses.

## RESULTS

### Insurance accelerates economic recovery

**Fig. 1.**
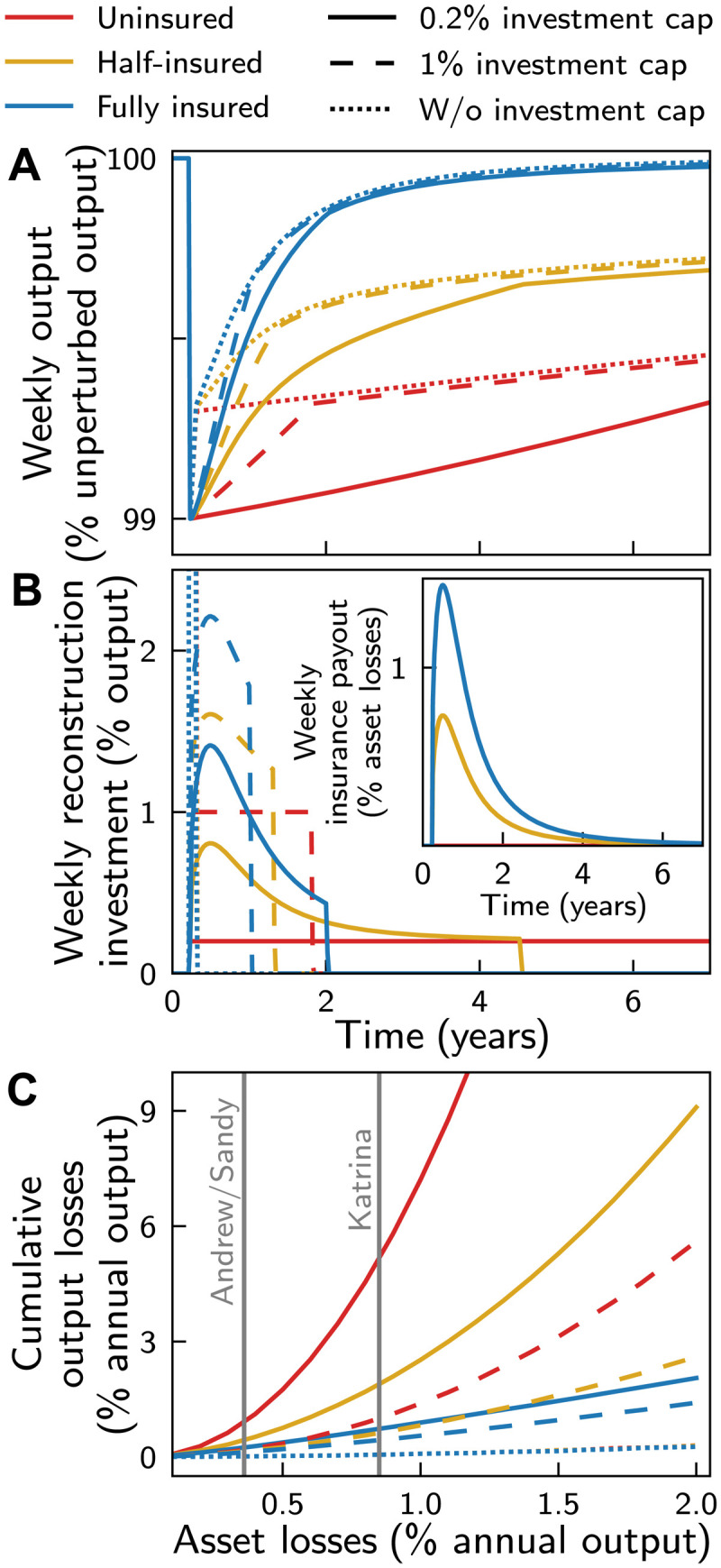
The contribution of insurance and reconstruction investment to the economic recovery dynamics in the aftermath of an individual hurricane with landfall. Response dynamics in the aftermath of a 1% shock to the stock of physical assets with no (red), 50% (ochre), and full (blue) insurance coverage, for scenarios where maximum weekly reconstruction investment is not limited (dotted lines) and limited to 0.2% (solid lines) and 1% (dashed lines) of weekly output, respectively. (**A**) Time series of weekly output relative to the output of an unperturbed economy on the BGP. (**B**) Time series of weekly reconstruction investment (in % of weekly output) and weekly insurance payout (in % of asset losses to the capital stock, inset). (**C**) Cumulative output losses until full recovery of production capacity as a function of the asset losses (both in terms of annual output in the year before the landfall). Gray vertical lines indicate the asset losses caused by the historical major hurricanes Sandy, Andrew, and Katrina according to the NatCatSERVICE database (*1*).

To illustrate the interplay of insurance payouts and limits of reconstruction investments, we first study the economic recovery dynamics in the aftermath of an individual storm that destroys 1% of the physical capital stock in month 3 (Fig. 1A). Besides the “realistic” standard calibration of the model (or scenario) (ochre full lines), we consider two limiting scenarios, one without insurance (red lines) and one with full insurance coverage of all losses (blue lines). Further, to test the sensitivity of the model with regard to the construction investment cap, we consider a 1% reconstruction investment cap (dashed lines) in addition to the 0.2% reconstruction investment cap (solid lines) [both caps were already used in (*40*)] and contrast both to a limiting case where all available investments (difference between output and savings) can be used for reconstruction (“no investment cap”, dotted lines) (Fig. 1B). Because insurance premiums depend on insurance coverage, each growth trajectory is normalized to the BGP of an unperturbed economy with the same insurance premium. To account for delays in insurance payouts, we fit data on cumulative insurance payouts of the Reinsurance Association of America (RAA) (*51*), indicating that 60% (90%) of the insured losses are paid out after 1 (3) year(s) with a sigmoidal function (Materials and Methods). The resulting weekly payouts are shown in the inset of Fig. 1B.

In line with empirical findings (*43*–*45*), recovery speed increases with insurance coverage for two reasons: First, because insurance provides additional financial means for reconstruction, the reconstruction investment cap can be temporarily exceeded, e.g., to compensate for scarcity driven wage increases (*52*). This accelerates the recovery process especially in the first reconstruction phase (see section S2 for a detailed discussion of the dynamics in this phase). Second, the larger the insurance coverage, the lower is the share of the output that has to be reinvested in reconstruction efforts. In consequence, more output can instead be invested in new capital fostering output growth especially in the slow recovery phase. Except in the limiting, overly optimistic, case of full insurance coverage and no reconstruction investment cap, cumulative output losses increase superlinearly with the size of the asset losses, i.e., indirect losses increase faster than shock size (Fig. 1C). In consequence, in the aftermath of intense hurricane shocks, it can take multiple months or even years for the economy to recover. For instance, in the standard scenario, it takes more than 5 months for the production capacity to recover after the major hurricanes Andrew and Sandy that struck Florida and Louisiana in 1992 and New York and New Jersey in 2012, respectively, both causing asset losses equivalent to about 0.4% of the U.S.’s annual output in the years of landfall, respectively (gray vertical lines in Fig. 1C). Further, our modeling suggests that in the aftermath of the largest historical loss event, the landfall of hurricane Katrina in New Orleans in 2005, which caused asset losses equivalent to 0.8% of the U.S.’s annual output in this year, it took more than 1 year and a half for the production capacity to recover.

### Growth losses increase with shock heterogeneity

**Fig. 2.**
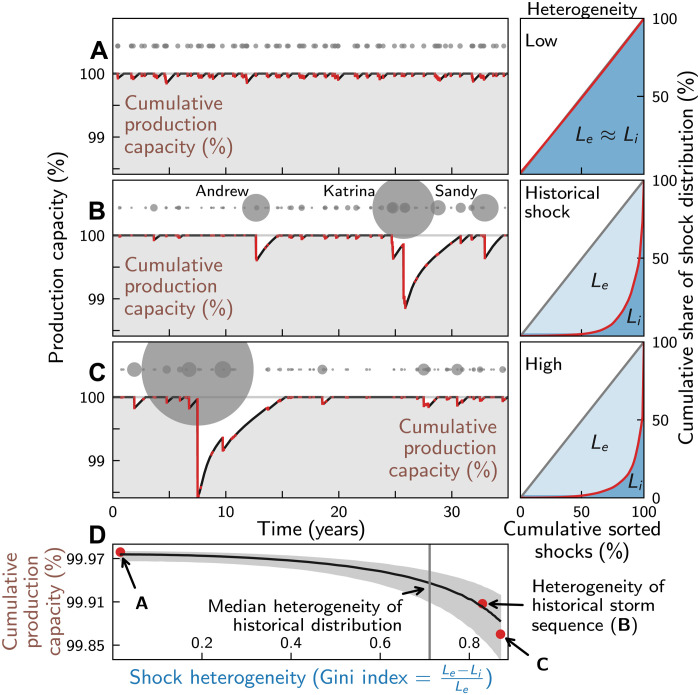
Recovery dynamics of production capacity in dependence of hurricane shock heterogeneity. Economic impacts of hurricane shocks for a period of 35 years. The heterogeneity of shocks increases from (**A**) to (**C**). Hurricane number and relative cumulative asset losses are fixed to the 88 hurricanes that reportedly made landfall in the United States over the period 1980–2014 and caused 3.24% of cumulative asset losses (relative to the GDP of the years the hurricanes made landfall) according to the NatCatSERVICE database (*1*). (**B**) Impacts of the observed historical time series of hurricanes with landfall. Left: Exemplary time series of available production capacity [in % of full production capacity (gray horizontal lines)]. Periods of reduced capacity in the disaster aftermaths are marked in red, and shocks are marked by gray dots with the size of the dots indicating the shock size. Right: Lorenz curves to illustrate the heterogeneity of the shock distribution. Red lines indicate the cumulative share of production capacity losses as a function of the cumulative share of the shocks. Gray diagonal lines indicate the Lorenz curves for equally distributed shocks. The Gini index $G\equiv \frac{L_{e}-L_{i}}{L_{e}}$ as measure for shock heterogeneity is determined by the ratio of the areas under the red (*L_e_*, light blue shading) and blue lines (*L_i_*, dark blue shading). (**D**) Mean cumulative available production capacity (in % of the production capacity of unperturbed system) as a function of the Gini index. Red dots and gray shaded areas indicate the values of the Gini index obtained for the runs in (**A**) to (**C**) and the 16.7 to 83.3 percentile confidence intervals, respectively. The gray vertical line indicates the median Gini index of the historical shock distribution (Materials and Methods). Other parameters: Insurance coverage 50% and reconstruction investment cap 0.2% of weekly output.

Next, we study how the economic response dynamics depends on the heterogeneity of hurricane shocks (Fig. 2). For that, we assume that (i) the number of landfall events is independent and Poisson distributed, and (ii) hurricanes have the same probability to make landfall in each month of the season (June to November). We do not account for potential clustering of hurricanes in event-rich periods, because it is currently unclear whether or to what degree such a clustering (overdispersion) has occurred in the historical period in near the U.S. coastline; while two early studies found evidence for overdispersion (*13*, *14*), a recent study suggests that these findings may, at least partially, have resulted from observational inhomogeneities in the storm data (*15*). After correcting for these reporting biases, the authors find no evidence for overdispersion in the Gulf of Mexico and near the U.S. East Coast. Further, we assume that asset losses (relative to the real national GDP in the year of landfall) are log normally distributed (see fig. S6 for a log-normal fit of the data). This yields conservative estimates of direct damages as even power law distributions with higher tail risk are currently discussed for U.S. hurricane damages (*53*, *54*).

In the remainder of this paper, we will refer to the distribution of relative asset losses as shock distribution. As detailed in Materials and Methods, drawing from this shock distribution allows us to generate synthetic time series of asset losses with defined length, event number, and value for the cumulative relative asset losses. To isolate the impact of shock heterogeneity, we then vary the heterogeneity of the direct asset losses—measured by the Gini index (*G*) of the event distribution—but keep the number of hurricanes with landfall (88) (and thus average hurricane return frequency) and the relative cumulative asset losses (3.24%) at their values reported in the NatCatSERVICE database (*1*) fixed for the study period 1980–2014 (35 years). Considering relative asset losses allows us to generate representative synthetic time series irrespective of the year of occurrence of each underlying event. Note that we thereby adjust losses for inflation and economic growth but assume no changes in vulnerability (e.g., due to adaptive measures taken on the ground) [see (*55*, *56*) for a discussion on different normalization approaches with respect to hurricane damages].

For a nearly homogeneous shock distribution (*G* = 0.018), asset losses (gray circles in Fig. 2, A to C) are relatively small, and production capacity can mostly recover between loss events and stays close to the one of the unperturbed system for the whole study period (Fig. 2A). For higher values of the Gini index, we obtain many small but few high-intensity loss events. Because cumulative output losses increase disproportionally with event intensity (cf. Fig. 1C), overall losses and the risk for incomplete recovery between events increase for higher values of the Gini index (cf. Fig. 2, B and C for *G* = 0.83 and *G* = 0.87). For instance, when driving the model with the historical sequence of landfall events, we find that the U.S. economy may not have recovered in between the major hurricanes Katrina and Sandy (Fig. 2B).

To gain a systematic understanding on how production capacity depends on shock heterogeneity, we study the cumulative production capacity over 35 years as a function of shock heterogeneity. For a given shock distribution, cumulative production capacity in general differs between event realizations due to differences in the timing and the size of the shocks. To account for this uncertainty, we generate a large ensemble of 20,000 realization for each shock distribution. The cumulative production capacity is then plotted as a function of the median Gini index as obtained across all realizations (Materials and Methods) (Fig. 2D). [Note that values of the Gini index for individual realizations may substantially deviate from the median Gini index. For instance, the Gini index for the observed historical storm sequence (*G* = 0.83) is substantially higher than the median value of the Gini index across all realizations for the historical storm distribution (*G* = 0.71) (compare red dot to gray vertical line in Fig. 2D).] We find that the available production capacity reduces superlinearly with increasing shock heterogeneity. The reduction is strongest in the high heterogeneity range to the right of the median Gini index for the historical period (gray line in Fig. 2D), where incomplete recovery becomes more likely.

**Fig. 3.**
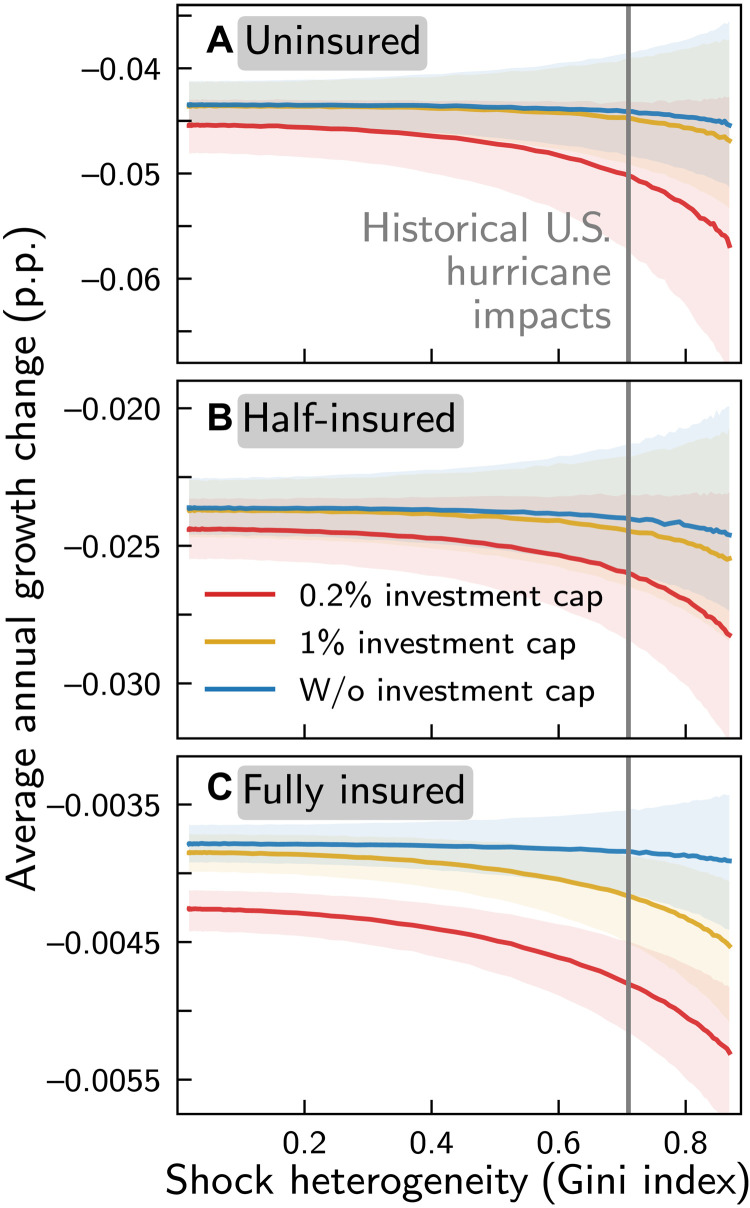
Impact of hurricane shock heterogeneity on annual output growth rate. Average annual growth rate change of the economy under hurricane shocks relative to the growth rate of the corresponding unperturbed economy, as a function of shock heterogeneity—measured by the Gini index—for no (**A**), half (**B**), and full (**C**) insurance coverage. Blue, ochre, and red lines depict median growth rate changes for scenarios where reconstruction investment is not limited, limited to 1%, and 0.2% of weekly output, respectively; shaded areas mark the corresponding 16.7 to 83.3 percentile confidence intervals. The gray vertical line indicates the median Gini index of the historical distribution of relative asset losses.

Similarly, economic growth declines superlinearly with increasing shock heterogeneity (Fig. 3). Besides the standard scenario with a 0.2% reconstruction investment cap and 50% insurance coverage (red line in Fig. 3B), we again consider scenarios with a 1% and no investment cap (ochre and blue lines in Fig. 3) as well as the limiting cases of no and complete insurance (Fig. 3, A and C). We find that the dependence of economic growth on shock heterogeneity increases when (i) the reconstruction investment cap and (ii) the insurance coverage are lowered.

For low values of the investment cap, the growth reduction with increasing shock heterogeneity can be quite substantial. For instance, for the standard scenario, median average annual growth losses increase by more than 16% from 0.0238 p.p. for the lowest to 0.0275 p.p. for the highest value of the Gini index (red line in Fig. 3B). While these growth rate reductions may appear small, they imply that for the highest value of the Gini index output losses accumulate over three and a half decade to US$ 16,218 per capita, an additional US$ 2196 per capita compared to the lowest value of the Gini index. The dependence of growth on shock heterogeneity can again be understood by the disproportional increase of indirect losses with shock intensity making incomplete recovery between events more likely with increasing Gini index (cf. Fig. 1A). In line with this reasoning, we find that, in the scenario without reconstruction investment cap, where the recovery time is substantially shorter than in the scenarios with caps (cf. Fig. 1), growth losses are nearly independent of the Gini index.

Further, for each fixed level of shock heterogeneity, growth losses decrease with increasing insurance coverage, which can be understood as follows: Insurance provides additional financial means for reconstruction and thereby mitigates the impact of shocks that are large compared to the reconstruction investment cap by reducing the recovery time and therefore suppressing incomplete recovery. For instance, for the standard scenario and the median Gini index of the historical period (gray vertical line in Fig. 3), output losses accumulate over three and a half decades to US$ 14,904 per capita. They are therefore, on average, US$ 832 per capita and US$ 1121 per capita higher than for the corresponding scenarios with a 1% and without reconstruction investment cap, respectively.

The greatest benefit of insurance is, however, that it strongly mitigates the magnitude of growth losses. For the median Gini index of the historical period and the lowest investment cap, hurricanes reduce the annual growth, on average, by 0.048 p.p. in the uninsured scenario. These losses are already roughly halved to 0.025 p.p. for the standard scenario with 50% insurance coverage and reduced by a magnitude larger than 10 to 0.0045 p.p. in the fully insured scenario. Accordingly, output losses accumulate over three and a half decade decrease from US$ 28,807, over US$ 14,904, to US$ 2746 per capita.

Critically, there is a trade-off between the increase in consumption in the disaster aftermath in the presence of insurance and consumption and economic growth losses due to lower capital accumulation in normal times. We find that the studied insurance scheme only fosters economic growth (figs. S8 and S9) and national consumption when large indirect losses arise, i.e., when the reconstruction process is slow and shocks are heterogeneously distributed as this likely was the case in the historical period. Thereby, the benefit of insurance for national consumption (averaged over many shock realizations) increases with insurance penetration and shock heterogeneity (figs. S10 and S11). Insurance premiums increase with insurance coverage but remain small compared to average per-capita consumption. For instance, for the standard scenario of 50% insurance and the mean shock heterogeneity of the historical period, the mean annual insurance premiums per capita equal US$ 110, which is only a tiny fraction (about 0.003%) of U.S. households’ average annual consumption in the historical period (fig. S12).

To set all these numbers into context, it is important to keep in mind that our model, by construction, computes growth losses borne by the United States in total. Local growth losses in the affected counties may be much larger.

### Better insurance coverage can help mitigate climate change–induced growth losses

**Fig. 4.**
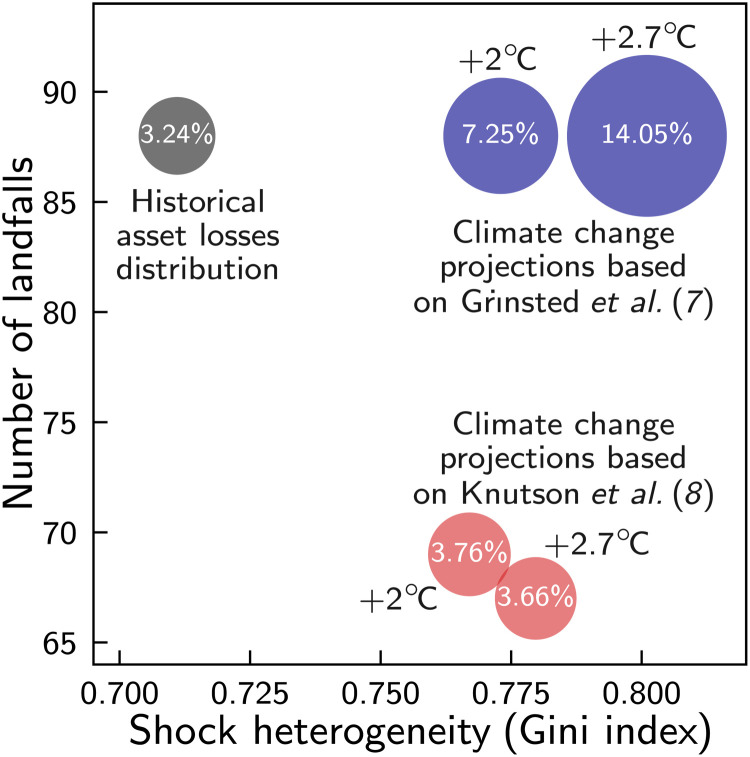
Visualization of climate change–induced shifts of the hurricane shock distribution. Under global warming, the historical distribution of the asset losses caused by the *N*_s_ = 88 historical hurricanes that made landfall in the United States in the 35-year period from 1980 to 2014 (black filled circle) according to the NatCatSERVICE database (*1*) is projected to change along three dimension: (i) the median shock heterogeneity measured by the Gini index (*x* axis), (ii) the number of landfalls for a 35-year period (*y* axis), and (iii) the median cumulative asset losses (size of circles). Blue and red circles indicate estimates for +2°C and + 2.7°C worlds (above preindustrial levels) based on Grinsted *et al.* (*7*) and Knutson *et al.* (*8*), respectively. The numbers in the circles refer to the median cumulative relative asset losses for a 35-year period (Materials and Methods).

To account for the substantial uncertainty on how climate change will affect on hurricane climatology, we use two different approaches, one at the lower and the other at the upper end of the impacts reported in the recent literature (*6*). Both approaches consistently predict an increase of the proportion of intense storms, although the magnitude of this change—and in consequence the resulting changes to asset losses—differs substantially between the two approaches. In contrast to the last section, where only the heterogeneity of events was mutable, these climate change–induced frequency increases may additionally translate into changes of the distribution of asset losses with respect to (i) the number of hurricanes and (ii) the cumulative asset losses during the study period (Fig. 4) (Materials and Methods). Knutson *et al.* (*8*) report a moderate increase of the return frequency of the most intense (categories 4 and 5) hurricanes by 45% in a 2°C world (2.7°C: 39%) but a reduction of the overall number of hurricanes (of all categories) by 22% (2.7°C: 24%), which the authors derive from changes in the maximum lifetime wind speeds of the storms obtained from dynamical downscaled global circulation model runs (“wind speed–based” estimate). In contrast, Grinsted *et al.* (*7*) use observational storm surge data and estimate a considerable increase of relative return frequencies ranging from 1.4-fold (2.7°C: 1.6-fold) for storms with a small surge index to a 6.4-fold (2.7°C: 15.2-fold) for the most intense storms (“surge-based” estimate) (*7*). The authors’ statistical analyses cannot distinguish whether this frequency increase is caused by an overall increase in the number of storms or merely implies a shift of the distribution of storm surges to higher intensity events. However, because there is relatively good agreement in the literature that the average number of hurricane per season will not strongly change with global warming (*6*), in our derivation of future asset losses according to Grinsted’s surge-based estimate, we assume that the number of storms does not change compared to the historical study period (Materials and Methods).

For both, the wind speed– and surge-based estimates, we obtain a moderate increase of shock heterogeneity with the median Gini index increasing from its historical value of 0.71 to 0.77 (2.7°C: 0.78) and 0.77 (2.7°C: 0.80), respectively. For the former, the storm number decreases to 69 (2.7°C: 67), whereas it remains unchanged (88) for the latter. Further, under the assumption of constant adaptation levels, the estimated cumulative relative asset losses over 35 years increase only moderately from 3.24% for the historical period to 3.76% (2.7°C: 3.66%) for the wind speed–based estimate but more than double (7.25%) (2.7°C: 14.05%) for the surge-based estimate.

**Fig. 5.**
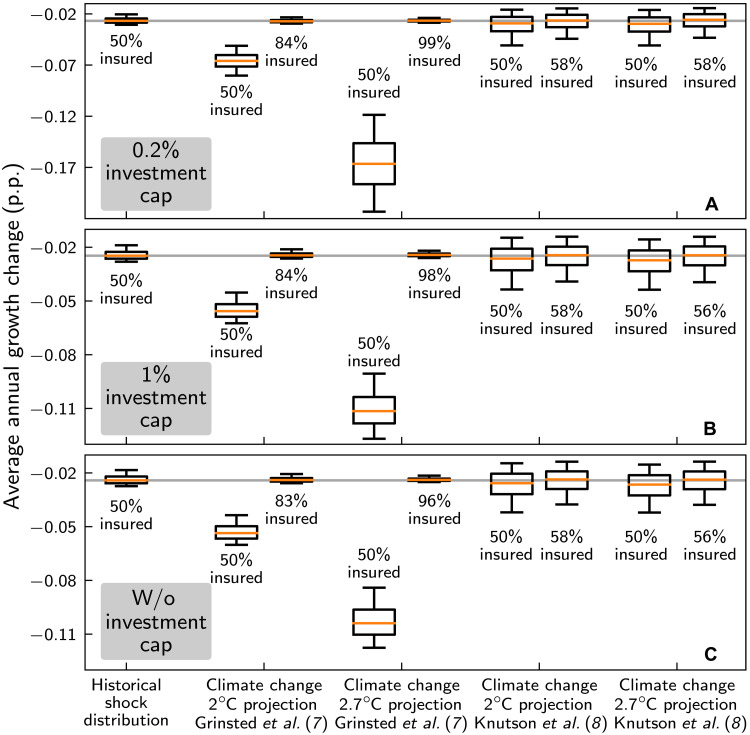
Projected impacts of hurricanes on economic growth in 2° and 2.7°C worlds and the effectiveness of insurance as coping strategy. Average annual growth losses (relative to the corresponding unperturbed economies evolving on the BGPs) as obtained for the historical shock distribution (50% insurance coverage, period 1980–2014; first column), for Paris-compatible +2°C warming above preindustrial levels (second, third, sixth, and seventh columns), and for +2.7°C warming in compliance with current policies (fourth, fifth, eighth, and ninth columns) for reconstruction investment caps of 0.2% (**A**) (standard scenario), 1% (**B**), and without reconstruction investment cap (**C**). Climate change projections of growth losses are derived from two different methods to estimate climate change–induced changes in the return frequencies of hurricanes by Grinsted *et al.* (*7*) and Knutson *et al.* (*8*) (50% insurance coverage, second and fourth columns, respectively). In addition, for both estimates and warming levels, the insurance coverages that would be necessary to reduce growth losses to the historical level are shown (third, fifth, seventh, and ninth columns). Orange lines, boxes, and whiskers indicate median loss estimates as well as the 25th to 75th and 5th and 95th percentile ranges, respectively.

In terms of median average annual growth losses, we obtain a moderate increase by 10% (for 2° and 2.7°C) compared to the historical standard scenario for the wind field–based estimate but a strong increase by 146% (2.7°C: 522%) for the storm surge–based estimate (Fig. 5A). The reason is that, for the former, the additional growth losses due to the increases of shock heterogeneity and cumulative asset losses are partially compensated by the reduction of growth losses due to the reduced absolute number of hurricanes; whereas for the latter, the increases of shock heterogeneity, cumulative asset losses, and hurricane number all enhance growth losses. Because we always consider growth losses relative to a baseline scenario with the same reconstruction investment cap and insurance coverage, these findings are robust with regard to changes in the reconstruction investment cap (cf. Fig. 5, B and C).

Regarding the question whether an increase in insurance coverage would be sufficient to compensate for the additional global warming–induced growth losses, we find that, to this end, the historical insurance coverage of 50% would have to be substantially raised to 84% (2.7°C: 99%) according to the surge-based estimate, whereas a moderate increase to 58% for 2°C and 2.7°C would suffice according to the wind field–based estimate for the standard scenario [cf. columns 2 and 6 with columns 3 and 7 (2.7°C: 5 and 9) in Fig. 5]. Again, these findings are fairly robust with regard to different values of the construction investment cap.

Last, we assess the debt levels which the insurance can temporarily accumulate when several severe storms make landfall in the United States within a short period of time and find them to be moderate. In the historical period, peak debt levels remain below 1.6% of the insured capital and, even for the future climate change impact scenario with the highest losses (+2.7°C and storm surge–based estimate of asset losses), peak debt levels remain below 5.6% of the insured capital for the standard calibration of the model [50% insurance coverage, 0.2% investment cap, and the median event heterogeneity of the historical (future projected) storms] (fig. S5). These peak debt levels are rarely reached, and in 95.5% of the simulations, debt levels remain below 0.9 and 3.3% of insured capital in the historical period and for the climate change impact scenario with the highest losses, respectively.

## DISCUSSION

These numbers suggest that a better insurance coverage could indeed be a viable means to compensate for climate change–induced increases in tropical storm–related losses, even in the absence of other adaptation measures. However, we caution that we do not account for several drivers of losses in the future projections, which may lead to an over- or underestimation of future losses. On the one hand, we assume no future changes in the vulnerability of the U.S. economy to hurricane impacts. While this might result in an overestimation of future losses, because vulnerability may be reduced by additional adaptation efforts, there also exists empirical evidence that the vulnerability of the U.S. economy to hurricane strikes has rather increased over the past decades (*57*, *58*). Assuming constant vulnerability thus provides a balanced perspective. On the other hand, our estimates of climate-induced changes in asset losses are based on estimates for the changes in storm number and the proportion of intense storms only; other potential channels through which climate change may affect on the economic losses caused by tropical storms, such as increasing storm surge risk due to sea level rise and stronger tropical cyclone precipitation rates (*6*), are neglected. Neglecting these additional drivers and noneconomic losses such as lives lost most likely results in an underestimation of future economic losses (*59*). Further, in our simulations, we do not consider that clusters of intense hurricanes may form in event-rich periods, because it is still discussed in the literature whether and to which extent this has happened in the historical period near the U.S. coastline (*13*–*15*). Clustering renders incomplete recoveries more likely, and these increase cumulative production capacity losses (Fig. 2D) and growth losses (Fig. 3). However, we find that even for an extreme and unprecedented scenario where all storms cluster within half of the 35-year study period, the increases in growth losses are small compared to the mitigating effect of higher insurance coverage (fig. S13). Because also the nonlinear dependence of growth losses upon shock heterogeneity remains robust, we may conclude that, even if the clustering of hurricanes will intensify under future global warming, the main findings of this work remain robust.

Further, using a simple macroeconomic growth model with only one homogeneous output good, our analysis cannot provide information on the recovery dynamics of individual sectors and may therefore underestimate delays arising from the scarcity of intermediate goods from strongly affected sectors needed for production in other sectors and the associated scarcity-induced price inflation in the disaster aftermath. In consequence, we may underestimate recovery costs (*60*) and, in turn, growth losses.

Our modeling framework assumes that continuous exponential economic growth will still be possible in the future period, as it was in the historical study period 1980–2014. This is in agreement with the Shared Socioeconomic Pathways (SSPs) mapping out a broad space of alternative socioeconomic futures and assuming that all national economies continue to grow exponentially at least until the end of the 21st century (*61*). In the light of dwindling and overused natural resources (*62*) and intensifying climate change impacts (*63*) (explicitly not accounted for in the SSPs), it can however not be taken for granted that continued growth is possible within the planetary boundaries (*64*). Thus, it would be an insightful extension of the current work to exchange the exponential growth model by a logistic model accounting for resource constraints (*65*) to assess the resulting impacts of hurricanes on growth and the effectiveness of the discussed insurance scheme. Testing the robustness of our modeling results for a large range of baseline growth rates, we find that insurance remains effective even under degrowth conditions (fig. S7). However, it is important to keep in mind that our modeling framework cannot account for the deep societal and economic transformations that would be required to allow for prosperity in a post or degrowth environment (*66*, *67*), including the acquisition of the necessary financial resources for the insurance scheme.

For the studied public and compulsory insurance scheme, we consider the risks of solvency issues or bankruptcy to be low because even under the higher warming scenario and for the more pessimistic storm surge–based estimate of asset losses, peak debt levels remain likely too low to severely burden the government’s budget (fig. S5). However, in most countries, insurance is currently provided either by competing private providers or a mixture of public and private providers (*68*), and solvency and indebtedness issues of providers have been reported in several countries (*19*, *47*). Further, insurance coverage and the access to insurance are usually more limited than in the studied insurance scheme (*16*). For instance, the U.S.’s NFIP is available only in participating communities (*19*, *46*). Further, because most insurance schemes are not mandatory, insurance take-up by the population can be limited because insurance premiums are perceived as too expensive (*24*) or are even unaffordable for poorer parts of the population (*69*). This problem of underinsurance may further aggravate in the future due to an increase of insurance premiums to compensate for the intensification of extreme weather events under global warming (*70*). Last, we do not discuss moral hazard issues that may arise from the considered mandatory precautionary savings scheme and may require the introduction of deductibles, for instance, to deincentive the construction of new buildings in storm surge–prone locations (*71*). For all these reasons, the insurance scheme discussed here likely provides an optimistic upper limit for the efficacy of insurance in mitigating disaster.

Our research stresses the importance of nonlinear economic responses to consecutive extreme weather events. In particular, our results suggest that only by (i) resolving the response to individual events and by (ii) accounting for a realistic timing of the events (e.g., accounting for the hurricane season), it is possible to estimate the full economic impact of extreme events (*28*). Further, these findings are key to assess the efficacy of adaptation and coping strategies. For instance, in our study, the limited pace of insurance payouts delays reconstruction efforts in the disaster aftermath, but a similar reasoning holds for physical protection measures such as sea walls or levees, which once breached may take months or even years to be repaired (*72*). Thus, temporally resolving the economic recovery phase is critical for the assessment and comparison of disaster response measures. This aspect becomes especially important because extreme weather events are projected to intensify and become more frequent with global warming, at least on a regional level (*5*). In this regard, our findings may also encourage the climate integrated assessment modeling community to consider new approaches allowing going beyond smooth damage functions translating changes in GMT into aggregate output losses. As shown here, this common approach may underestimate the economic repercussions of extreme weather events because it neglects potentially important nonlinearities in the economic response such as the disproportional increases of indirect losses with impact intensity or the case of incomplete recovery (*28*). This may also explain the discrepancy between the loss estimates reported in the recent climate econometrics literature and the estimates of climate IAMs.

While our estimates on how climate change may affect on economic losses caused by hurricanes in the United States are subject to several sources of uncertainty, they nonetheless show that the mitigating effect of increased insurance coverage is of the same order of magnitude as the climate change–induced loss increase. Although insurance premiums may increase under global warming by up to a factor of four, they likely will remain affordable for U.S. consumers. This suggests that insurance can be a major building block of future climate change adaptation strategies, at least in developed countries. For developing countries, the hurdles to adapt to climate change are much higher because they are often more strongly affected by—and more vulnerable to—climate change impacts and lack the financial means and strong institutions to implement comprehensive climate adaptation measures (*73*). To illustrate this, we have analyzed the hurricane-prone Small Island Developing State of Haiti (see section S1) and find that the hurricane-induced growth losses it suffers in the present climate are already by one magnitude larger than those of the United States (cf. Fig. 3B with fig. S16A). One reason is that Haiti’s disaster insurance market is much less developed, and nearly all of the past hurricane losses were not insured (*1*). Further, already in the present climate, Haiti is affected so strongly that even in the idealistic limit of full insurance coverage, it would still suffer growth losses comparable in magnitude to those of the United States today (cf. Fig. 3B with fig. S16C), and hurricane impacts are projected to further aggravate for Haiti under continued climate change (fig. S17), at least according to the more pessimistic storm surge–based damage projection. To this end, our results stress the importance—for developing and developed countries alike—to complement insurance solutions with other measures to build resilience to extreme weather events such as investments into better housing standards and resilient infrastructure (*74*) or coping strategies such as managed retreat (*75*) in a risk-layering approach (*76*). However, in contrast to rich developed countries of the Global North, strongly affected developing countries will be only able to successfully adapt to climate change impacts when national and international mechanisms and institutions providing concessional climate finance and expertise in climate adaptation such as the United Nations’ Green Climate Fund are further strengthened by ensuring that they have both the financial resources and the effective government to fulfill their mandates.

## MATERIALS AND METHODS

### Modeling approach

As the standard neoclassical Solow-Swan growth model for a closed economy (*77*), our Insured Growth under Climate Impacts model (InGroClIM) describes the growth of a per-capita stock of physical capital *k* for a unique indistinguishable good under investments and capital depreciation. Here, we neglect changes in labor market and population growth as drivers of capital growth. In extension to the standard model, we account for a nonprofit insurance scheme and obtain two coupled differential equations for *k* and the per-capita capital stock of the insurance *k*_I_

$$\dot{A}=\text{Λ}A\;\;\;\;$$ (1A)

$$\dot{k}=sy-[\delta +r_{I}]k+F_{I}$$ (1B)

$${\dot{k}}_{I}=r_{I}k-F_{I}\;\;\;$$ (1C)

Here, $\overset{\cdot }{\left(\cdot \right)}$ denotes the derivative with respect to time *t*. We assume that total factor productivity (TFP) *A* grows exponentially with trend growth rate Λ, and *s*, *y*, and δ denote savings rate, production function, and depreciation rate of capital, respectively. The insurance premium *r*_I_ ≡ *r*_I_(*r*_c_) depends on the economy’s insurance coverage *r*_c_, and *F*_I_(*t*) denotes the insurance payouts in the disaster aftermaths. Both terms are detailed below. Further, we assume in (Eq. 1B) that the production process can be described by a Cobb-Douglas production function *y*(*t*) ≡ *A*(*t*)(*k*(*t*))^α^, where α ∈ (0,1] denotes the capital share of income. We model the impact of extreme weather events as shocks to the capital stock. Following (*40*), we assume that the reconstruction of destroyed capital provides higher marginal returns than investment into new technologies. This allows us to describe the economic recovery in the disaster aftermath as the superposition of two different mechanisms: (i) a fast reconstruction process of the damaged capital and (ii) the comparatively slow growth of the capital stock due to technological development. To this end, we write the capital stock as the product of the fraction of remaining production capacity ξ(*t*) ∈ [0,1] and a “potential capital stock” *k*_p_*(t)*

$$k(t)\equiv \xi (t)k_{p}(t)$$ (2)

The Cobb-Douglas production function is derived from the assumption that the process of capital accumulation is optimal and that the last unit of capital added is the least productive (*78*). However, it appears unlikely that a disaster strikes in such a way that it “deconstructs” the capital in the same optimal way, starting with the least productive unit, and this method is likely to underestimate direct production losses [see discussion in (*41*) for details]. Following previous works (*40*, *41*), we therefore assume that a shock does not merely destroy the least efficient capital but equally affects all productivity layers of capital. For that, we may write *y* as a function of ξ and *k*_p_

$$y(t)\equiv y(\xi (t)\;\&\;k_{p}(t))=\xi (t)A{\left(k_{p}(t)\right)}^{\alpha }$$ (3)

Noteworthy, this implies that at the time of the shock *t*_s_, *y* reads

$$y(t_{s})=\xi (t_{s})\lim_{t↗t_{s}}[y(t)]=\xi (t_{s})A{\left(k_{p}(t_{s})\right)}^{\alpha }$$

where ξ(*t*_s_) < 1, and *k*_p_(*t_s_*) represents the predisaster value of the capital stock. Thus, production is reduced by the same factor 1 − ξ(*t*) as the capital stock, i.e., asset losses equal direct production losses, and the marginal productivity of capital remains unchanged.

To derive the dynamical equations for *k*_p_ and ξ, we first decompose the total investment *I(t)* into the sum of two different investment channels: short-term reconstruction investments *I*_ξ_(*t*) and regular investments increasing production capacity *I_k_*(*t*)

$$I(t)\equiv sy(t)+F_{I}(t)=I_{k}(t)+I_{\xi }(t)$$ (4)

By using Eqs. 2 to 4, we may then rewrite the dynamical equation for the capital stock (Eq. 1B) as

$$(\xi (t){\dot{k}}_{p}(t))=\dot{\xi }(t)k_{p}(t)+\xi (t){\dot{k}}_{p}(t)\;\;$$ (5A)

$$\;\;\overset{\mathrm{Eq}.(1B)}{=}I_{k}(t)+I_{\xi }-[\delta +r_{I}]\xi (t)k_{p}(t)$$ (5B)

By comparing the right-hand sides of Eqs. 5A and 5B, we obtain the dynamical equations for *k*_p_ and ξ as

$${\dot{k}}_{p}(t)=\frac{I_{k}(t)}{\xi (t)}-[\delta +r_{I}]k_{p}(t)$$ (6A)

$$\dot{\xi }(t)=\frac{I_{\xi }(t)}{k_{p}(t)}\;\;\;\;$$ (6B)

Next, we derive an expression for *I*_ξ_(*t*), which then permits us to calculate *I_k_* from Eq. 4. To this end, we have to make four assumptions: First, we assume that reconstruction investments yield higher returns compared to investments in the potential capital stock and are therefore prioritized. Second, we assume that reconstruction efforts are limited by short-term constraints such as a lack of skilled labor or reconstruction materials, which may substantially slow down the economic recovery. In consequence, only a fraction *f*_max_ ∈ [0,1] of the output available for investment *sy*(*t*) can be used to finance reconstruction; the actual value of the investment cap *f*_max_ depends on the economy under consideration (*40*). Third, we assume that reconstruction efforts cease when the capital stock equals the potential capital stock, no overshoot is possible. Last, we assume that the insurance primarily finances reconstruction efforts. In the presence of insurance, the investment cap may be temporarily exceeded because the insurance provides additional financial means, e.g., to compensate for scarcity driven wage increases (*52*). This assumption is motivated by empirical findings that higher insurance coverage can lead to a faster economic recovery (*73*). However, if reconstruction is completed before all of the insured capital is reimbursed, then the remaining insurance payout will be invested into the potential capital stock. With these assumption, we may express *I*_ξ_(*t*) as

$$I_{\xi }(t)\equiv \{\begin{array}{cc} 0 & \xi (t)=1\\ \min[\min[f_{\max},s]y(t)+F_{I}(t),I_{r}(t)] & \xi (t)<1 \end{array}$$ (7)

where *I_r_*(*t*) ≡ (1 − ξ(*t*))*k*_p_(*t*) is the investment needed to reconstruct the capital stock in the present time step.

### Insurance payout dynamics

We model insurance as a compulsory precautionary savings mechanism, which may be implemented and managed on the national level by a public institution. To our knowledge, there are no empirical data on the payout dynamics of such an insurance scheme in the United States. This is why we use observational data of insurance payouts of commercial providers of risk diversifying insurance by the RAA (*51*), arguing that the payouts dynamics of the insurance scheme discussed here and commercial (re-) insurers may be similar to main processing steps such as the filing of insurance claims and that their eligibility assessment by the insurance provider would be identical for both insurance schemes. According to the RAA data, the reimbursement of insured losses *f_I_*(*t*) can spread over several years; 60% (90%) of the insured values are reimbursed within 1 (3) year(s). This may substantially delay the reconstruction process. We describe the cumulative insurance payouts with a sigmoidal function

$$\begin{array}{rl} & f_{I}(t-t_{s};r_{c}\Delta_{s}k_{p}(t_{s}))\\ & \;\equiv r_{c}\Delta_{s}k_{p}(t_{s})\beta \frac{{\left(\frac{t-t_{s}}{\tau_{I}}\right)}^{\beta -1}(a-1)\exp[-{\left(\frac{t-t_{s}}{\tau_{I}}\right)}^{\beta }]}{\tau_{I}{\left(1+(a-1)\exp[-{\left(\frac{t-t_{s}}{\tau_{I}}\right)}^{\beta }]\right)}^{2}}\;\forall t>t_{s} \end{array}$$

Here, *t*_s_ denotes the time of the shock, and the insured losses are given by the product of the insurance coverage *r*_c_, the asset loss Δ_s_ at time *t*_s_ relative to the preshock potential capital stock *k*_p_(*t*_s_). (Note that, according to Eq. 3, this is identical to expressing asset losses relative to the output in the year before the shock as done for the calibration of the model to empirical data). The three parameters *a*, τ_I_, and β (*51*) are specified in Table 1 (see fig. S2 for a fit of the observational data). The cumulative insurance payout in response to multiple successive asset losses {Δ_s*_i_*_}*_i_* at times {*t*_s*_i_*_}*_i_* is then given by the sum of the individual payouts

$$F_{I}(t;{\left\{t_{s_{i}}\right\}}_{i},{\left\{\Delta_{s_{i}}\right\}}_{i})\equiv \sum_{i=1}^{N_{s}}f_{I}(t-t_{s_{i}};r_{c}\Delta_{s_{i}}k_{p}(t_{s_{i}}))$$

where index *i* labels the shock number and *N*_s_ denotes the total number of shocks.

**Table 1.** Exogenous parameters used in the numerical simulations.

Quantity	Symbol	Value	Unit
Initial GDP per capita	*y* ^0^	51638.1	US$
GDP growth rate	*g*	2.6%	Year^−1^
Savings rate	*s*	0.2	Year^−1^
Capital depreciation rate	δ	0.1	Year^−1^
Capital share of income	α	0.7	
Time step length	Δ*t*	$\frac{1}{52}$	Year
Insurance payout parameter one	*a*	10^9^	
Insurance payout parameter two	β	0.0741	
Insurance payout parameter three	τ_I_	1.31 x 10^−18^	Year
Empirical insurance premium coefficient	ε	4.046 x 10^−4^	
Simulation period	𝒯	35	Year
Cumulative relative historical asset losses	Δ_𝒯_	3.24	%
Number of historical landfalling hurricanes	*N*_s_	88	
Standard deviation of historical log-normal asset loss distribution	σ_0_	0.10654	

### Model calibration

We assume that, in the absence of shocks, the economy evolves along its BGP, where output growth is constant and only driven by TFP growth (growth rate Λ)

$$g\equiv \frac{\dot{y}}{y}=\frac{\dot{A}}{A}+\alpha \frac{\dot{k}}{k}=\Lambda +\alpha g\;⟺\;\Lambda =(1-\alpha )g$$ (8)

In the second identity, we have used that, if *y* grows constantly with rate *g*, then *k* also grows constantly with the same rate. This can be seen as follows: From the first identity in Eq. 8, it follows that the growth rate of the capital stock $\frac{\dot{k}}{k}=\frac{g-\Lambda }{\alpha }$ is constant when *g* is constant. From Eq. 1B, it then follows that *k* and *y* have to grow with the same rate *g*. Because in the absence of shocks *F*_I_(*t*) = 0 ∀ *t* ∈ [0, 𝒯], where 𝒯 denotes the length of the simulation, the dynamic equations for *k* and *k*_I_ decouple (cf. Eq. 1), it suffices to solve the equations of motions for the dynamic variables *A* and *k* along the BGP. The corresponding equation for *k*_I_ can then be derived from Eq. 1C. To this end, we insert the coordinate transformation

$$A(t)=e^{\Lambda t}\tilde{A}(t)\;\&\;k(t)=e^{gt}\tilde{k}(t)$$

into the dynamic equations for *A* and *k* yielding

$$\dot{\tilde{A}}(t)=0\;\;\;\;\;\;\;$$ (9A)

$$\dot{\tilde{k}}(t)=s\tilde{y}(t)-(\delta +r_{I}+g)\tilde{k}(t)$$ (9B)

where we have introduced the output in BGP coordinates $\tilde{y}(t)\equiv A^{0}{\tilde{k}}^{\alpha }(t)$ with A^0^ denotes initial TFP. Equating the right-hand sides of Eqs. 9A and 9B to zero yields the steady states for *A* and *k* in BGP coordinates

$${\tilde{A}}^{⋆}=A^{0}\;\&\;{\tilde{k}}^{⋆}=k^{0}={\left(\frac{sA^{0}}{\delta +r_{I}+g}\right)}^{\frac{1}{1-\alpha }}$$ (10)

where (·)^⋆^ the steady-state values of variables and *k*^0^ denote the initial capital stock. This allows writing the BGP solution of Eqs. 1 as

$$A(t)=e^{\Lambda t}A^{0}\;\;\;\;\;$$ (11A)

$$k(t)=e^{gt}k^{0}\;\;\;\;\;\;$$ (11B)

$$k_{I}(t)=\frac{r_{I}}{g}[k(t)-k^{0}]=\frac{r_{I}}{g}k^{0}[e^{gt}-1]$$ (11C)

To calibrate the model to the United States, we set the initial per-capita annual output *y*^0^ and output growth rate *g* to the per-capita GDP and the GDP growth rate of the United States in 2015 according to the World Banks’ and Organization for Economic Co-operation and Development’s National Accounts database (https://data.worldbank.org/indicator/NY.GDP.PCAP.CD), whereas capital depreciation rate δ, savings rate *s*, and capital share of income α are set to their standard values for developed economies (*77*). Using the Cobb-Douglas relation for the production function *y* = *Ak*^α^ and the steady-state relation for *k*^0^ (cf. Eqs. 10A and 10B) then allows expressing the initial TFP and initial per-capita stock as $A^{0}=y^{0}{\left(\frac{\delta +r_{I}+g}{s}\right)}^{\alpha }$ and *k*^0^ = *sy*^0^(δ + *r*_I_ + *g*)^−1^, respectively.

Table 1 lists all exogenous parameters used in the simulations. Note that our model results are very robust with regard to changes of the GDP growth rate *g* because we only consider changes of the perturbed economy relative to an unperturbed economy evolving along the BGP. Even large variations of *g* ∈ [−1 % ,4 %] result in changes of growth losses that are small compared to the climate uncertainties (compare lines and shaded areas in fig. S7).

Modeling a nonprofit insurance scheme, we have to ensure that, averaged over many realizations, the insurance does neither make profit nor losses. However, deriving an exact analytical formula for the corresponding insurance premium *r*_I_ is challenging because—as output losses and growth losses—it would depend on shock heterogeneity. Instead, we here motivate a simple heuristic formula neglecting this dependence and show that the resulting average insurance profits or losses are negligible compared to the cumulative payouts of the insurance. In the worst case, the total relative asset losses occur at the last time step of the simulation. Covering this loss would require an insurance capital stock of *k*_I_(𝒯) = *r*_c_Δ_𝒯_*k*(𝒯), where 𝒯 denotes the length of the simulation. Inserting this relation in the BGP solution for *k*_I_ (cf. Eq. 11) provides us with the following expression for the insurance premium

$$r_{1}\equiv \epsilon \frac{{gr}_{c}}{1-e^{-gT}}$$

where we have added an empirically determined factor ε, ensuring that average insurance profits (or losses) are negligible. (cf. fig. S4 revealing that average profits or losses of the insurance are about five magnitudes smaller than the insured capital.)

### Gini index as measure for shock heterogeneity

We fit the relative asset losses of the *N*_s_ = 88 historical hurricanes with landfall included in the NatCatSERVICE database (*1*) (cf. table S1) with a log-normal distribution (fig. S3) with standard deviation (SD) σ_0_. To change the heterogeneity of the loss events, we vary the SD σ of the log-normal distribution from $\frac{\sigma_{0}}{100}$ to 4σ_0_. We use the Gini index $G\equiv \frac{L_{e}-L_{i}}{L_{e}}\in [0,1]$ as measure for the shock heterogeneity, which is derived from the difference of the areas below the Lorenz curves for a uniform distribution *L_e_* and the given shock distribution *L_i_* (cf. Fig. 2). Shock heterogeneity increases from small to large values of the Gini index. Noteworthy, the Gini index of the historical time series of hurricanes with landfall equals 0.829, whereas the median Gini index of the historical shock distribution—obtained by averaging over many synthetic realizations of asset loss time series (see the next section for details)—equals 0.71.

### Generation of synthetic time series of asset losses

In this section, we discuss the generation of synthetic time series of asset losses from their historical distribution as reported by the NatCatSERVICE (*1*) database and the global data set of tropical cyclone exposure (TCE-DAT) (*49*). For the study period 1980–2014 of 𝒯 = 35 years, these databases list *N*_s_ = 88 hurricanes with landfall that have caused asset losses corresponding to at least 10^−4^ % of the GDP in the year of their landfall (see table S1). Over this period, relative asset losses accumulated to Δ_𝒯_ = 3.24%. We generate synthetic time series of asset losses of length 𝒯 keeping *N*_s_ and Δ_𝒯_ at their historical values in three steps illustrated in fig. S6. First, following (*15*), we assume that the number of hurricanes with landfall *n_a_* in each season *a* is Poisson distributed, $f_{P}(n_{a})\equiv \frac{\lambda^{n_{a}}e^{-\lambda }}{n_{a}!}$. Further, we assume that the mean number of landfalls per season λ is constant over the study period 𝒯. To ensure that each synthetic track contains exactly *N*_s_ shocks, the shock number for the last season of the track is set to the remainder of available shocks $N_{s}-\sum_{a=1}^{T-1}\;n_{a}$. To avoid that the last season always receives the remainder of available shocks, seasons are shuffled afterward. Second, we assume that the likelihood of a hurricane making landfall is the same for each day of the season, but exclude the possibility that two hurricanes make landfall at the same day. Third, following (*53*), we assume that relative asset losses Δ_s_ are log normally distributed, $f_{\mathrm{LN}}(\Delta_{s})\equiv \frac{1}{s\Delta_{s}\sqrt{2\pi }}\exp\left[-\frac{{\left(\ln(\Delta_{s})-m\right)}^{2}}{2s^{2}}\right]$ (cf. fig. S3), where we have introduced the parameters $s\equiv {\left(\ln\left(\frac{\sigma^{2}}{\frac{\Delta_{T}^{2}}{N_{s}}}+1\right)\right)}^{\frac{1}{2}}$, $m\equiv \ln(\frac{\Delta_{T}}{N_{s}})-\frac{s^{2}}{2}$, and the SD σ of the log-normal distribution. Similarly, first, the size of the last shock of each realization is set to the difference between Δ_𝒯_ and cumulative relative asset losses before the last shock to ensure that total cumulative relative asset losses equal Δ_𝒯_, and then shock sizes are reshuffled.

### Storm surge– and wind field–based climate change projections of asset losses

#### Storm surge–based projections of asset losses

Grinsted *et al.* (*7*) estimated the relative increase in the average return frequency of hurricanes with landfall in dependence of the severity of their storm surge [measured by the surge index (*79*)] per degree of GMT warming relative to the reference period 1980–2000. We use these findings to project asset losses for +2^∘^C and +2.7^∘^C increases of GMT above its preindustrial level. [Note that 1°C of global warming compared to 1980–2000 corresponds to 1.5^∘^C of warming compared to the preindustrial level (*5*)]. To this end, we first map the surge indices $\left\{\int_{s_{i}}^{h}\right\}$ of the *N*_s_ = 88 historical hurricanes that made landfall in the United States between 1980 and 2014 to the corresponding relative asset losses $\left\{\Delta_{s_{i}}^{h}\right\}$ reported in the NatCatSERVICE database (table S1) (*1*). Next, we determine the statistical correlation between historical asset losses and surge indices, yielding the damage function *f*(*s*) (fig. S14). As discussed in the Results, we assume that the average number of hurricanes with landfall will not change compared to the historical study period. In consequence, we interpret the increases in return frequency reported by Grinsted *et al.* (*7*) as increases solely in storm surge intensity and not as an increase of the average number of hurricanes making landfall (in each season). This allows us to map the set of historical surge indices $\left\{\int_{s_{i}}^{h}\right\}$ to a set of estimated surge indices in a warmer world $\left\{\int_{s_{i}}^{cc}\right\}$. We then assume that each future relative asset loss ${\text{Δ}}_{s_{i}}^{cc}$ can be written in terms of the corresponding historical asset loss. This allows expressing future relative asset losses in terms of the historical relative asset losses as well as future and historical storm surge indices

$${\text{Δ}}_{s_{i}}^{cc}\equiv {\text{Δ}}_{s_{i}}^{h}+f\left(\int_{s_{i}}^{cc}\right)-f\left(\int_{s_{i}}^{h}\right)$$ (12)

Note that with this relationship, historical asset losses are reproduced for $\int_{s_{i}}^{cc}=\int_{s_{i}}^{h}$. Using Eq. 12, we project relative asset losses Δ_𝒯_ accumulated over 𝒯 = 35 years to increase substantially from their historical value of 3.24 to 7.25% in a +2^∘^C world (14.05% in a +2.7^∘^C world). We then generate synthetic realizations of future asset loss time series by distributing the projected *N*_s_ = 88 relative asset losses over the simulation time of 𝒯 = 35 years as described above.

#### Wind field–based projections of asset losses

Knutson *et al.* (*8*) analyzed an ensemble of downscaled global climate models participating in the fifth phase of the Coupled Model Intercomparison Project. On the basis of the wind fields of the storms, they estimated a median decrease of 22% in the overall number of all hurricanes but a median increase of the most intense category 4 and 5 storms by 45% for an increase of GMT by +2°C above its preindustrial level under the Representative Concentration Pathway 4.5. To estimate the associated changes in asset losses, we first divide the *N*_s_ = 88 historical tropical cyclones that reached land with at least tropical storm strength in the United States in the period 1980–2014 into moderate (categories 0 to 3, 66 storms) and intense (categories 4 and 5, 22 storms) storms. The definition of a storm’s landfall and the categorization of the storms by the Saffir-Simpson scale was done according to the TCE-DAT (*49*) and IBTRaCS (*80*) databases. The definition of a storm’s landfall and the categorization of the storms by the Saffir-Simpson scale was done according to the TCE-DAT database (*49*). Applying then the estimates of Knutson *et al.* (*8*), we project that in a +2°C (+2.7°C) world, the number of all hurricanes and the number of moderate hurricanes decrease to 69 and 37, respectively, whereas the number of intense hurricanes increases to 32. This would lead to a minor change of relative cumulative asset losses Δ_𝒯_ from their historical value of 3.24 to 3.76% (3.66%). Synthetic time series of future asset losses are lastly generated as described for the surge-based estimate.

## Supplementary Materials

### This PDF file includes:

Sections S1 and S2 Tables S1 and S3 Figs. S1 to S18

## Notes

**Clarification (2 February 2023):** In the Introduction and Materials and Methods subsection “Wind field-based projections of asset losses,” the authors have provided explanations for the way they categorized tropical cyclones as tropical storms and hurricanes and the definition of landfall. This information was added to further explain the methodology the authors used in their research. The calibration of the economic growth model does not depend on the categorization of the storms as it is based on hurricane damage data only. Due to the addition of this information, references in the main text and the Supplementary Materials have been renumbered.
