# Supplementary material for: Better insurance could effectively mitigate the increase in economic growth losses from U.S. hurricanes under global warming
Source: Sci Adv. 2023 Jan 4;9(1):eadd6616. doi: 10.1126/sciadv.add6616 (PMC9812378; doi:10.1126/sciadv.add6616)
Supplement: Supplementary file 1 — Sections S1 and S2 Tables S1 and S3 Figs. S1 to S18 [file sciadv.add6616_sm.pdf]

Science Advances
AAAS

Supplementary Materials for

Better insurance could effectively mitigate the increase in economic growth losses from U.S. hurricanes under global warming

Christian Otto *et al.*

Corresponding author: Christian Otto, christian.otto@pik-potsdam.de

Sci. Adv. **9**, eadd6616 (2023)
DOI: 10.1126/sciadv.add6616

This PDF file includes:

Sections S1 and S2
Tables S1 and S3
Figs. S1 to S18

S1 The case of the Small Island Developing State of Haiti

In this section, we repeat the modelling exercise described in the main text to the hurricane-prone Small Island Developing State of Haiti. According to MunichRe's NatCatSERVICE *(1)* database, Haiti experienced 18 tropical hurricanes with landfalls in the study period 1980–2014 (Tbl. S3). Asset losses (relative to the growth domestic product of the years the hurricanes made landfall) accumulate to 11.86%. After fitting them with a log-normal distribution (Fig. S15), we obtain a median Gini index of 0.58. Driving the model with the parameters of Tbls. 1 and S2, we find that, as for the US, Haitian growth losses increase nonlinearly with shock heterogeneity but decrease with insurance coverage (cf. Fig. 3 with Fig. S16. Noteworthy, according to NatCatSERVICE none of the hurricane damages were insured in the historical study period, compared to 50% in the US. Further, we assume the same insurance payout dynamics as for the US since there are no data available for Haiti.

Besides the higher direct losses, also the comparably smaller number of storms that made landfall in Haiti (18 historical storms compared to 88 in the US) may contribute to the differences in growth losses between the two countries. To test the contribution of the shock number to growth losses, we keep cumulative relative asset losses and shock heterogeneity at their historical levels for Haiti, but increase the number of shocks to 88. In principle, there are two counteracting mechanisms: On the one hand, a higher storm number increases the chance of incomplete recoveries which would lead to higher growth losses but, on the other hand, the size of the shocks decreases with increasing shock number which would reduce growth losses. We find that the second effect nearly balances the first, and growth losses increase only marginally when going from 18 to 88 shocks (Fig. S18). This leads us to the conclusion that the substantially higher growth losses for Haiti primarily result from the comparably higher direct losses and, only to a very small extent, from the lower shock number.

Future projections of damages

Due to the lack of better data and to use the same method, we assume for the storm surge-based estimate that the scaling of the return frequencies of hurricane-induces storm surges with global warming is the same as in the US. Further, assuming that the number of events remains unchanged compared to the historical study period, we find that cumulative relative asset losses increase to 13.89% and 17.60% for the +2°C and +2.7°C degree scenarios, respectively. Growth losses more than double and triple for the +2°C and +2.7°C degree scenarios, respectively (Fig. S17). According to the wind-speed based estimate, the total number of events declines to 14 landfalls for both, the +2°C and +2.7°C degree scenarios which results in moderate decreases of cumulative relative asset losses to 11.82% and 11.76%, respectively. In consequence, median growth losses also slightly decrease compared to the historical period (Fig. S17). As for the US, increasing insurance coverage allows mitigating the additional climate change induced growth losses

arising for the storm surge-based estimate. However, it is important to note that already in the historical period Haiti suffered growth losses which may be unsustainably high.

S2 Recovery dynamics in the fast recovery phase

In this section, we derive an analytical approximation for the recovery dynamics of the output in the first (fast) phase of the recovery. From Eq. (3), we see that the first derivative with respect to time of output y/y_0 (relative to the output of the unperturbed system y_0) can be written as

$$\dot{\left(\frac{y}{y_0}\right)} = \dot{\left(\frac{\xi k_p^\alpha}{\xi_0 k_0^\alpha}\right)} = \left(\dot{\xi} k_p^\alpha + \alpha \xi k_p^{\alpha-1} \dot{k}_p\right) k_0^{-\alpha} - \alpha \xi k_p^\alpha k_0^{-(\alpha+1)} \dot{k}_0 \tag{1a}$$

$$\overset{k_p \approx k_0}{\approx} \dot{\xi} + \frac{\alpha \xi}{k_0} \left(\dot{k}_p - \dot{k}_0\right). \tag{1b}$$

In the second equality of Eq. (1a), we have used that the output of the unperturbed system may be written as $y_0 = A k_0^\alpha$, where k_0 is the capital stock of this system. Further, in Eq. (1b), we have approximated the potential capital stock of the perturbed system by the capital stock of the unperturbed system which is strictly valid until the time of the shock but – in good approximation – remains valid throughout the fast recovery phase. From, Eq. (1b), we see that the recovery rate of relative output is described by the superposition of the recovery rate of the production capacity $\dot{\xi}$ and a term which is proportional to the product of ξ and the differences of the rates of change of $\dot{k}_p$ and $\dot{k}_0$. In the absence of insurance, (solid red lines in Fig. (1)), $\dot{\xi}$ is constant and equals the reconstruction investment cap $f_{\max}$ (cf. Eq. (7)) if sufficient investments are available. This is always the case for the comparably small shocks considered in this study. Further, for the fast time scale of the ξ recovery (first recovery phase), we may assume that the term $\dot{k}_p - \dot{k}_0$ is approximately constant since k_p and k_0 are slowly varying compared to the fast variable ξ. Thus, in the first recovery phase, the first term on the right-hand-side of Eq. (1b) is constant, while the second term increases exponentially when the production capacity is restored. This explains the somewhat concave shape of the output recovery curve. The insurance increases the recovery rate of the production capacity $\dot{\xi}$ and renders it time-dependent. Since in the disaster aftermath, insurance payouts first rapidly increase before they decay on a longer time scale, the output recovery curve assumes a convex shape.

Tbl. S1. Historical hurricanes that made landfall in the US between 1980 and 2014. 1st through 4th columns list names and years of landfall of the storms as reported by the IBTRaCS database *(80)*, storm severity (category 4-5 hurricanes according to Saffir-Simpsons scale), and storm surge index according to ref. *(79)*, respectively. The 5th column reports categorized asset losses based on reported asset losses by Munich Re's NatCatSERVICE database *(1)*: small ($> 10^{-4}\%$), moderate ($> 10^{-3}\%$), strong ($> 10^{-2}\%$), severe ($> 10^{-1}\%$). The asset losses are measured relative to the growth domestic product of the US (according to World Banks' and OECD's National Accounts database (`https://data.worldbank.org/indicator/NY.GDP.PCAP.CD`)) in the year of landfall.

Name	Year	Cat. 4-5 hurricane	Surge index	Asset losses category
Alberto	1994		9.2	strong
Alicia	1983		52.7	strong
Allen	1980	x	36.4	strong
Allison	1989		20.8	moderate
Allison	2001		24.1	strong
Andrew	1992	x	23.4	severe
Arlene	1993		11.5	small
Barry	2001		9.8	small
Bertha	1996		16.4	moderate
Beryl	1994		4.6	moderate
Bill	2003		9.4	small
Bob	1985		6.8	small
Bob	1991		3.4	strong
Bonnie	1986		14.6	small
Bonnie	1998		22.7	strong
Bonnie	2004		5.8	small
Bret	1999	x	4.7	moderate
Chantal	1989		11.9	moderate
Charley	2004	x	3.2	severe
Charley	1986		7.8	small
Charley	1998		12.7	moderate
Cindy	2005		1.9	moderate
Claudette	2003		55.1	moderate
Danielle	1980		2.6	small
Danny	1985		14.5	moderate
Danny	1997		19.1	moderate
Debby	2012		8.1	moderate
Dennis	2005	x	108.1	strong
Dennis	1981		5.6	small
Dennis	1999		5.3	moderate
Diana	1984	x	9.6	moderate
Dolly	2008		7.9	moderate

Name	Year	Cat. 4-5 hurricane	Surge index	Asset losses category
Earl	1998		22.7	small
Edouard	1996	x	2.6	small
Elena	1985		33	strong
Emily	1993		7.3	small
Erin	1995		30.3	moderate
Erin	2007		7.9	small
Ernesto	2006		7.2	moderate
Fay	2008		6.9	moderate
Florence	1988		9.2	small
Floyd	1999	x	17.6	strong
Fran	1996		4.4	strong
Frances	2004	x	9.4	strong
Frances	1998		25.8	moderate
Gabrielle	2001		5.4	moderate
Gaston	2004		5	small
Georges	1998	x	85.4	strong
Gilbert	1988	x	9.5	moderate
Gloria	1985		15.7	strong
Gordon	1994		6.1	moderate
Gordon	2000		7	small
Gustav	2008	x	71	strong
Hanna	2008		2.3	small
Hermine	2010		8.5	moderate
Hugo	1989	x	25.9	severe
Humberto	2007		7.9	small
Ida	2009		16.3	moderate
Ike	2008	x	105.1	severe
Iniki	1992		3.9	strong
Irene	1999		10.8	moderate
Irene	2011		55.6	strong
Isaac	2012		80.3	strong
Isabel	2003	x	8.1	strong
Iselle	2014		3.5	small
Isidore	1984		5.5	small
Isidore	2002		47.4	moderate
Ivan	2004	x	53	severe
Iwa	1982		2.5	moderate
Jeanne	2004		14	strong
Jerry	1989		6.8	small
Josephine	1996		10.1	moderate
Juan	1985		34.1	strong
Kate	1985		8.4	moderate
Katrina	2005	x	114.4	severe
Keith	1988		9.4	small
Lee	2011		14.9	strong

Name	Year	Cat. 4-5 hurricane	Surge index	Asset losses category
Lili	2002	x	5.6	strong
Marco	1990		6.7	small
Mitch	1998	x	5.1	moderate
Opal	1995	x	59.3	strong
Ophelia	2005		6.1	small
Paul	2006		13.7	small
Rita	2005	x	35.6	severe
Sandy	2012		15.7	severe
Tammy	2005		5.6	small
Wilma	2005	x	55.1	severe
?	1987		–	small

Tbl. S2. Exogenous parameters used in the numerical simulations for Haiti. Parameters not mentioned compared to Tbl. 1 were not modified compared to parameters for the USA.

Quantity	Symbol	Value	Unit
Initial GDP per capita	y^0	1402.1	US$
GDP growth rate	g	1.95%	year^{-1}
Cumulative relative historical asset losses	$\Delta_{\mathcal{T}}$	11.86	%
Number of historical landfalling hurricanes	N_s	18	
Standard deviation of historical log-normal asset loss distribution	σ_0	1.3909	

Tbl. S3. Historical hurricanes that made landfall in Haiti between 1980 and 2014. 1st through 4th columns list names and years of landfall of the storms as reported by the IBTRaCS database *(80)*, storm severity (category 4-5 hurricanes according to Saffir-Simpsons scale), and storm surge index according to ref. *(79)*, respectively. The 5th column reports categorized asset losses based on reported asset losses by Munich Re's NatCatSERVICE database *(1)*: small ($> 10^{-4}\%$), moderate ($> 10^{-3}\%$), strong ($> 10^{-2}\%$), severe ($> 10^{-1}\%$), devastating ($> 1\%$). The asset losses are measured relative to the growth domestic product of the US (according to World Banks' and OECD's National Accounts database[2]) in the year of landfall.

Name	Year	Cat. 4-5 hurricane	Surge index	Asset losses category
Allen	1980	x	36.4	devastating
Alpha	2005		-	moderate
Dean	2007	x	-	moderate
Dennis	2005	x	108.1	severe
Ernesto	2006		7.2	moderate
Georges	1998	x	6.9	devastating
Gilbert	1988	x	85.4	moderate
Gordon	1994		9.5	severe
Gustav	2008	x	6.1	moderate
Hanna	2008		71.0	moderate
Ike	2008		2.3	moderate
Irene	2011		105.1	moderate
Isaac	2012		55.6	strong
Jeanne	2004		80.3	severe
Noel	2007		14.0	strong
Olga	2007		-	moderate
Sandy	2012		15.7	devastating
Sandy	2008		-	small

[2] `https://data.worldbank.org/indicator/NY.GDP.PCAP.CD`

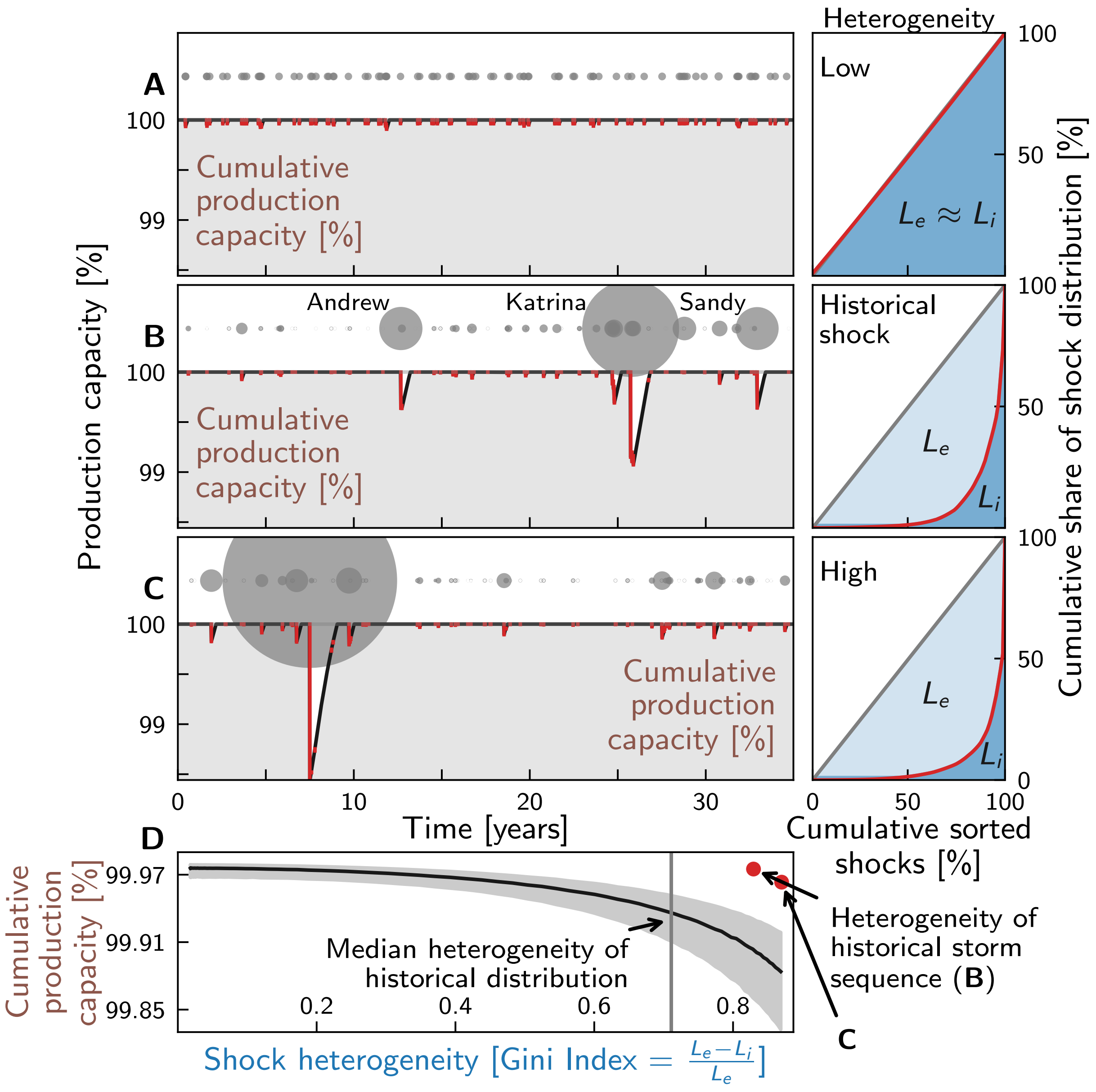

Fig. S1. Recovery dynamics of production capacity in dependence of shock heterogeneity for 1% reconstruction investment limit. Same as Fig. 2 but for a 1% reconstruction investment cap.

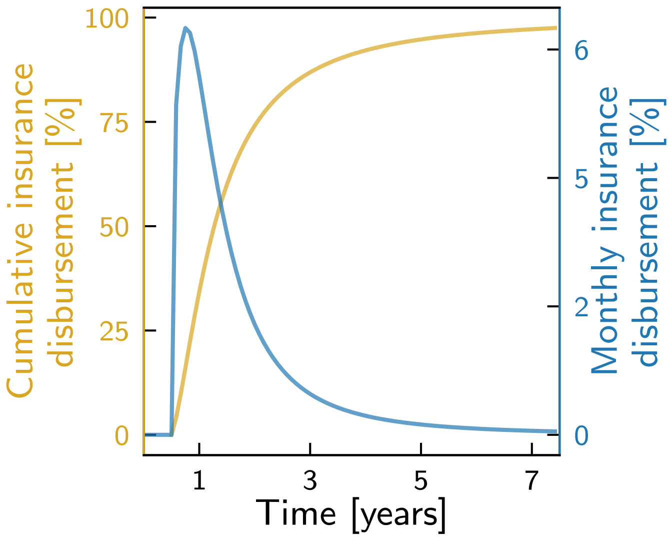

Fig. S2. Insurance payout dynamics. Cumulative (ochre) and monthly (blue) insurance payouts in the aftermath of an individual shock to the physical capital stock. The sigmoidal function for the cumulative payouts is calibrated such that 60% (90%) of the insured values are reimbursed within one (three) year(s) according to insurance data of the Reinsurance Association of America *(51)*. The monthly payouts are then obtained by deriving this function with respect to time.

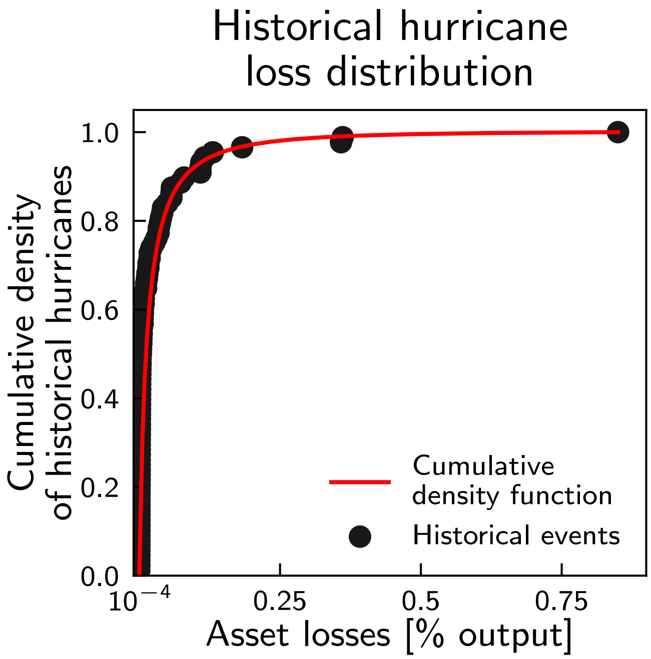

Fig. S3. Distribution of the historical asset losses of hurricanes that made landfall in the US in the period 1980–2014. Black dots depict the asset losses as reported by the NatCatSERVICE database *(1)* in the period 1980–2014 relative to the US growth domestic product of the years in which the hurricanes made landfall. The red line depicts a fit with a log-normal cumulative density function.

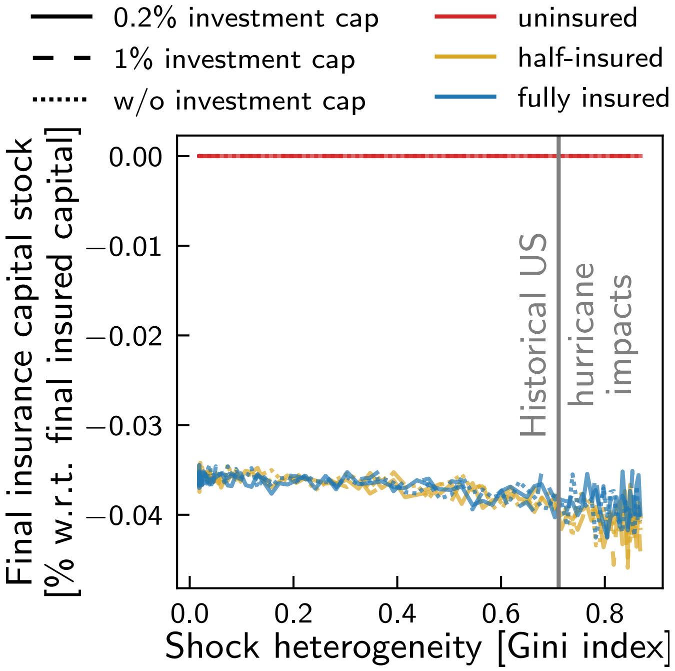

Fig. S4. Insurance is non-profit. Median insurance capital stock in terms of the insured potential capital stock after $\mathcal{T} = 35$ years. Same scenarios and colour code as in Fig. 1.

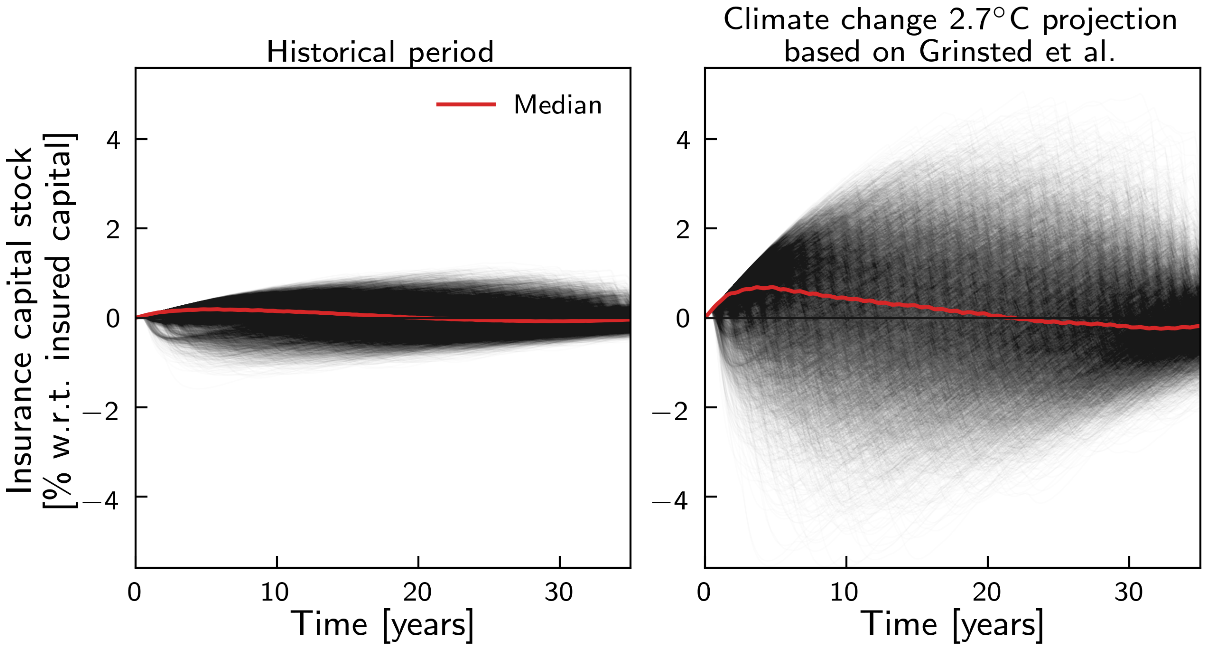

Fig. S5. Time series for insurance capital stock. Full ensemble of time series of insurance capital stock relative to the stock of insured capital for the main calibration of the model (50% insurance coverage as reported by NatCatService *(1)* and a reconstruction investment cap of 0.2% of weekly output) for the historical period (left) and for the future climate change impact scenario with the highest losses (+2.7°C and storm surge-based estimate of asset losses) (right). Each black line depicts one simulation run and the red lines indicate respective medians.

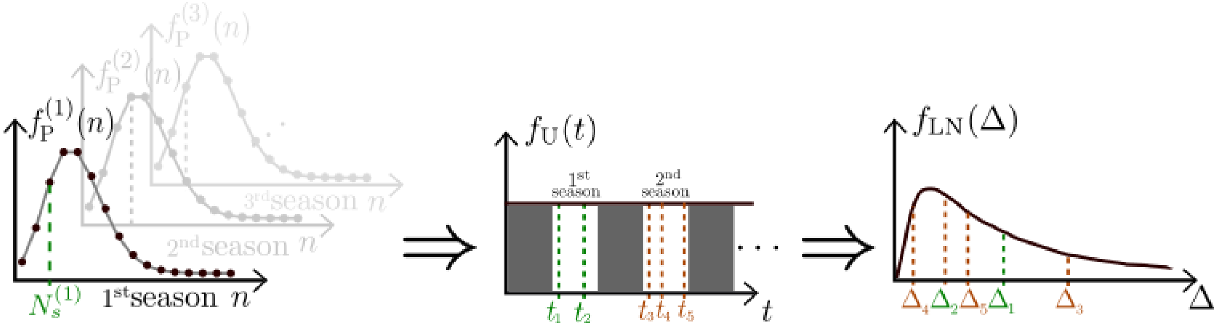

Fig. S6. Sketch of construction of synthetic asset loss time series caused by hurricanes with landfall. Synthetic time series of asset losses are generated in three steps: First, the number of hurricane shocks in each US hurricane season (June–November) is drawn from a Poisson distribution f_P. Second, the times of landfalls are determined assuming the same probability of landfall within each season, excluding the possibility of two landfalls on the same day. Third, the relative asset loss of each landfall is drawn from the log-normal distribution of Fig. S3.

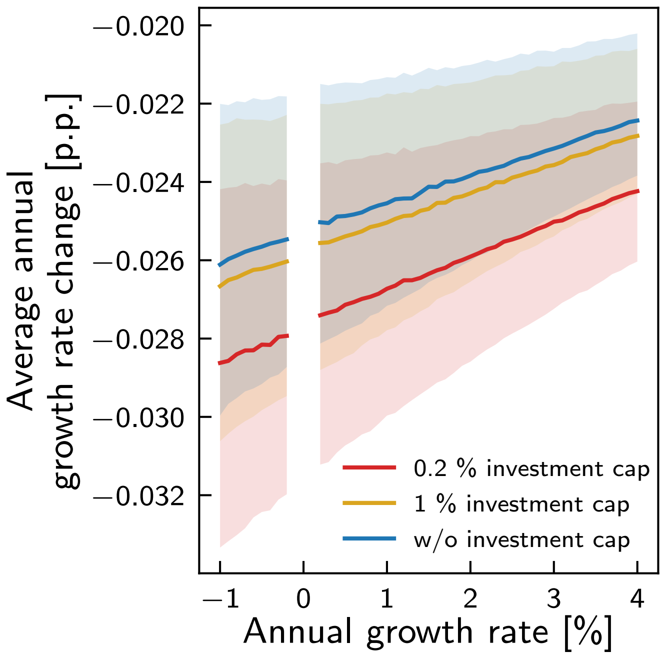

Fig. S7. Robustness of relative growth losses with regard to baseline growth rate. Dependence of average annual growth losses compared to the corresponding unperturbed baseline scenario for an insurance coverage of 50% without reconstruction investment limit (blue) as well as for reconstruction investment caps of 0.2% (red) and 1% (ochre) of weekly output, respectively. Lines indicate median growth rate reductions and shaded areas the corresponding 16.7–88.3 percentile confidence intervals.

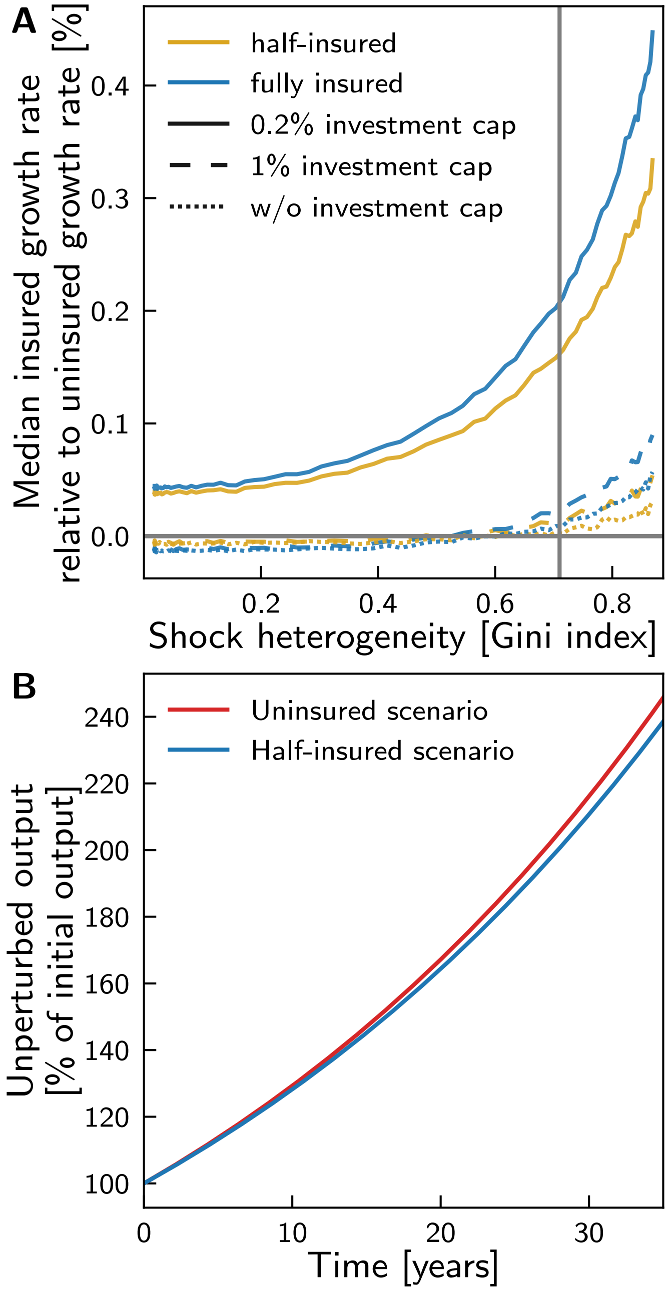

Fig. S8. Impact of insurance coverage on economic growth.
A: Change of median growth rate for half (ochre lines) and fully (blue lines) insured economies with regard to an economy without insurance in dependence of shock heterogeneity for reconstruction investments capped to 0.2% (solid), 1% (dashed) of weekly output and without investment cap (dotted). The vertical grey solid line denotes the median Gini index of the historical shock distribution. Parameters as in Tbl. 1.
B: GDP time series for an unperturbed and uninsured economy (red solid line) and an unperturbed but half insured economy (blue solid line) for the historical period of 35 years. Parameters as in Tbl. 1.

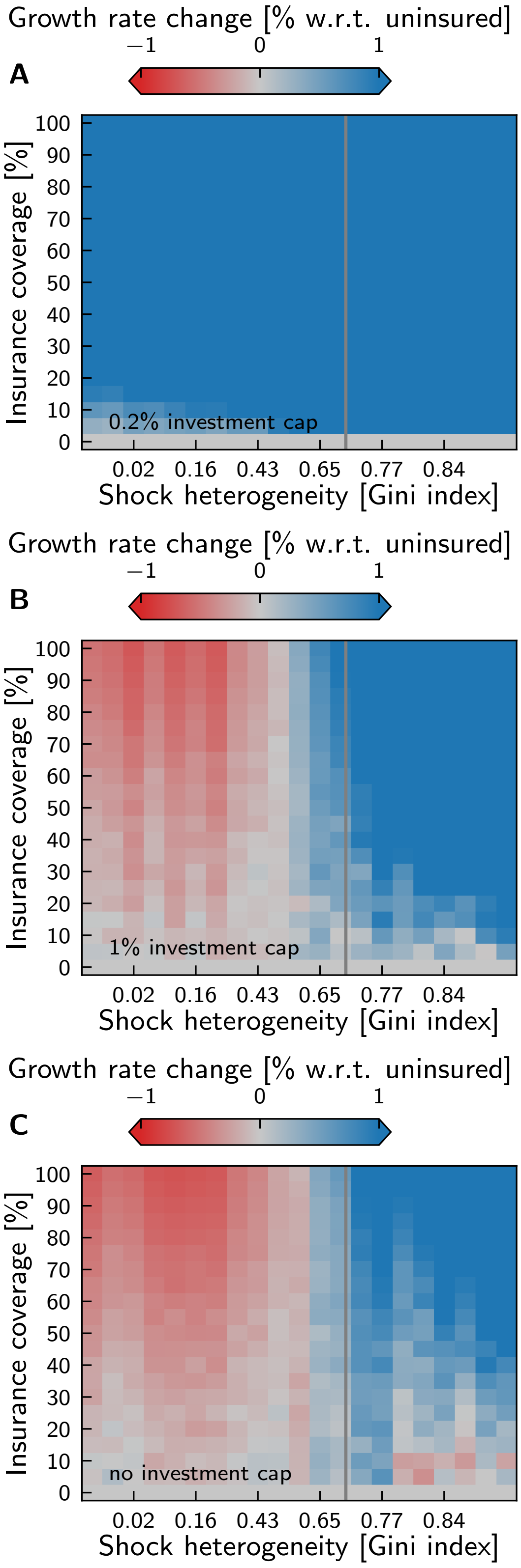

Fig. S9. Growth rate change in dependence of shock heterogeneity and insurance coverage. Parameters as in Tbl. 1. The grey vertical line indicates the median Gini index of the historical distribution of relative asset losses.

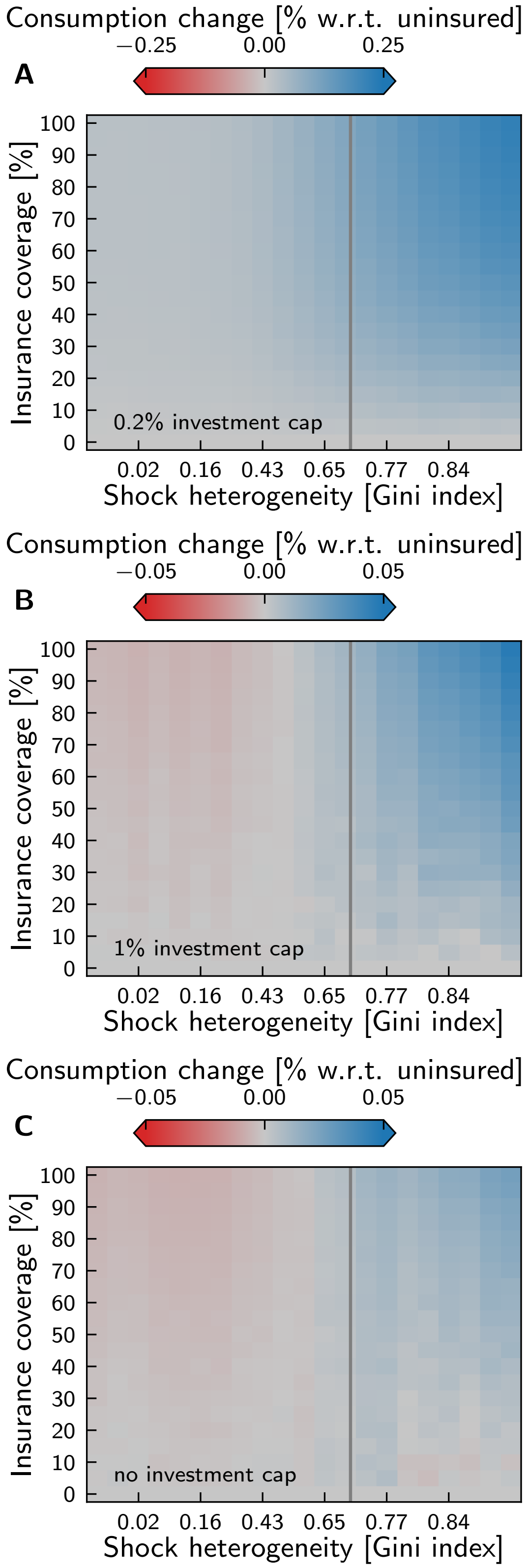

Fig. S10. Consumption change in dependence of shock heterogeneity and insurance coverage. Parameters as in Tbl. 1. The grey vertical line indicates the median Gini index of the historical distribution of relative asset losses.

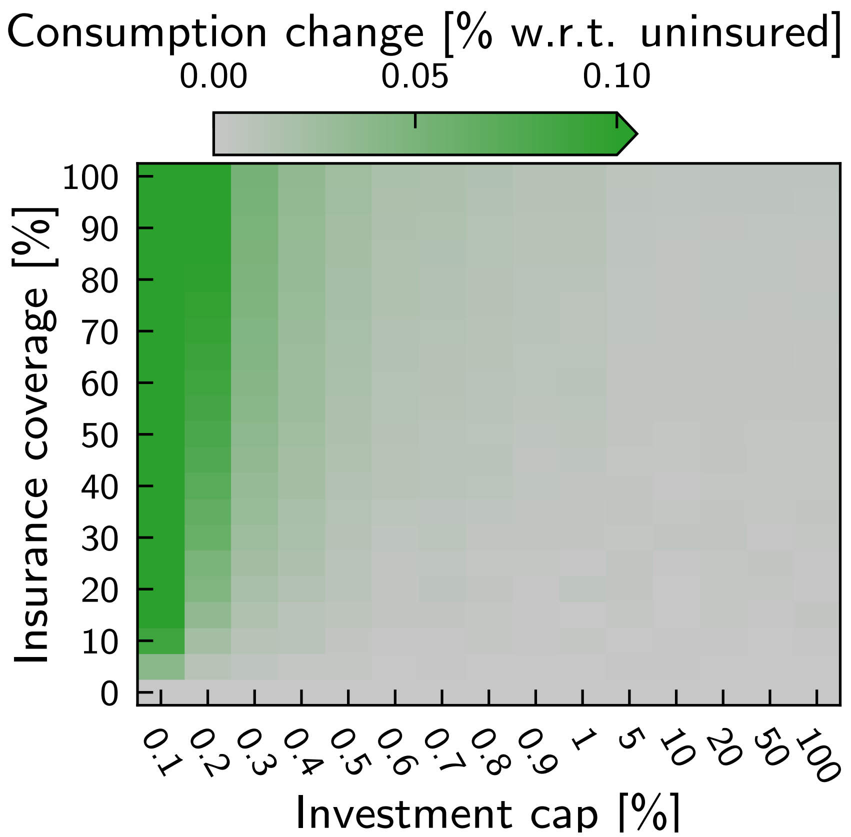

Fig. S11. Consumption change in dependence of insurance coverage and investment cap. Parameters as in Tbl. 1. The grey vertical line indicates the median Gini index of the historical distribution of relative asset losses.

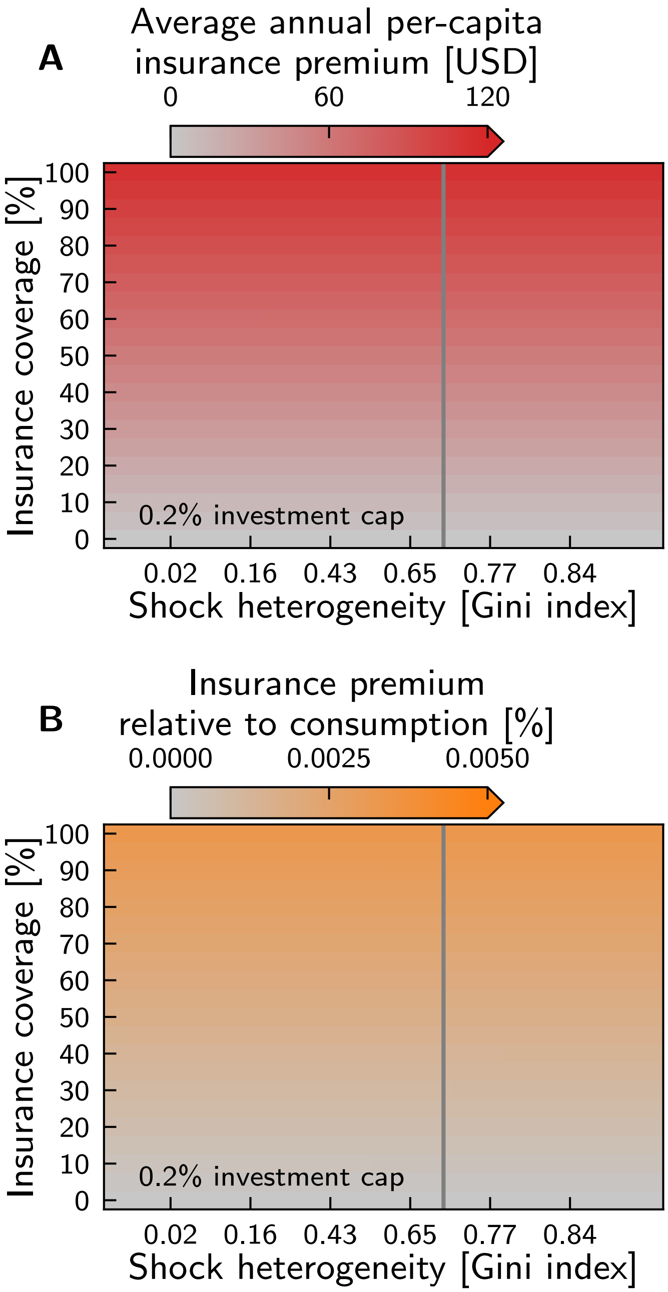

Fig. S12. Average annual insurance premium in dependence of shock heterogeneity and insurance coverage.
The grey vertical line indicates the median Gini index of the historical distribution of relative asset losses.
A: Absolute per-capita insurance premium in US$. **B**: Insurance premium relative to average consumption. Parameters: investment cap: 0.2% of weekly output; other parameters as in Tbl. 1.

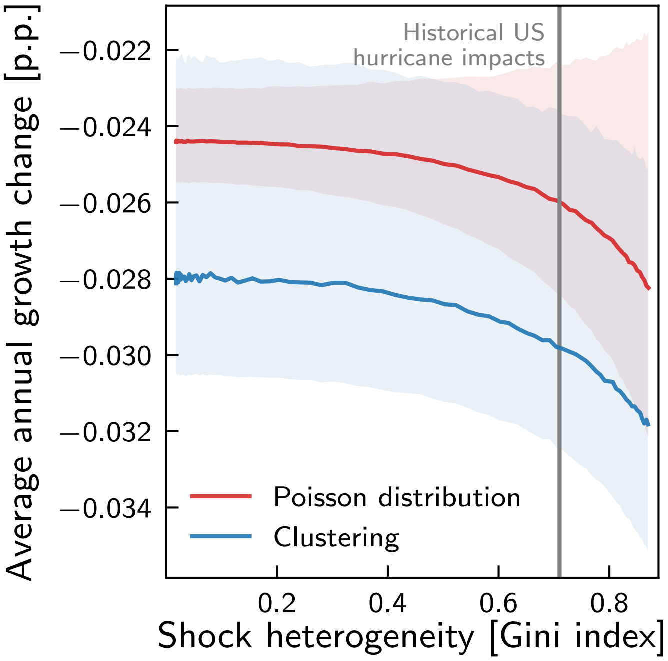

Fig. S13. Growth losses for independent and clustering hurricanes. Average annual growth losses compared to the corresponding unperturbed baseline scenario as a function of shock heterogeneity for independent (and thus Poisson distributed) hurricanes (red) and for an hypothetical clustering scenario where all 88 storms cluster within half of the 35 years study period (18 years) (blue). (The timing of this 18 year clustering period shifts randomly between the different realisations.) Lines indicate median growth rate reductions, shaded areas the corresponding 16.7–88.3 percentile confidence intervals, and the vertical grey solid line denotes the median Gini index of the historical shock distribution. Parameters: Main model calibration (50% insurance coverage and reconstruction investment capped to 0.2% of weekly output).

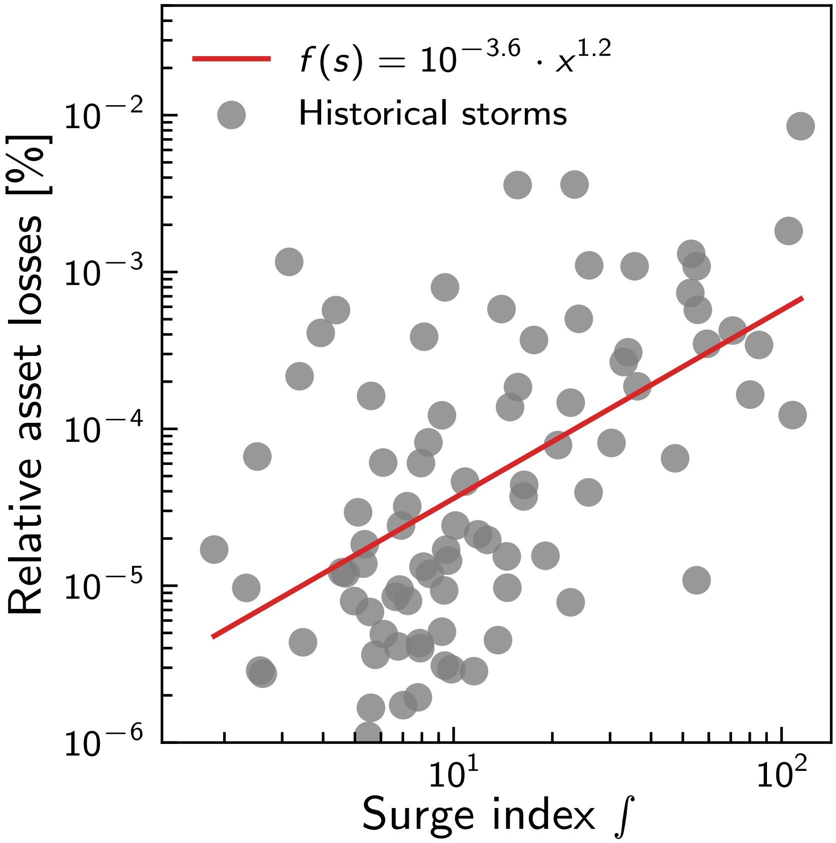

Fig. S14. Dependence of asset losses on surge index. Log-log plot of asset losses (grey dots) of the 88 historical hurricanes that made landfall in the US between 1980 and 2014 according to NatCatSERVICE database *(1)* relative to the growth domestic output the year of landfall (according to the World Banks' and OECD's National Accounts database (`https://data.worldbank.org/indicator/NY.GDP.PCAP.CD`) as function of their surge index *(79)*. The red line denotes a non-linear fit of the data (damage function $f(\int)$). The Pearson's chi-squared criteria for the goodness-of-fit is $\chi^2 = 0.59$).

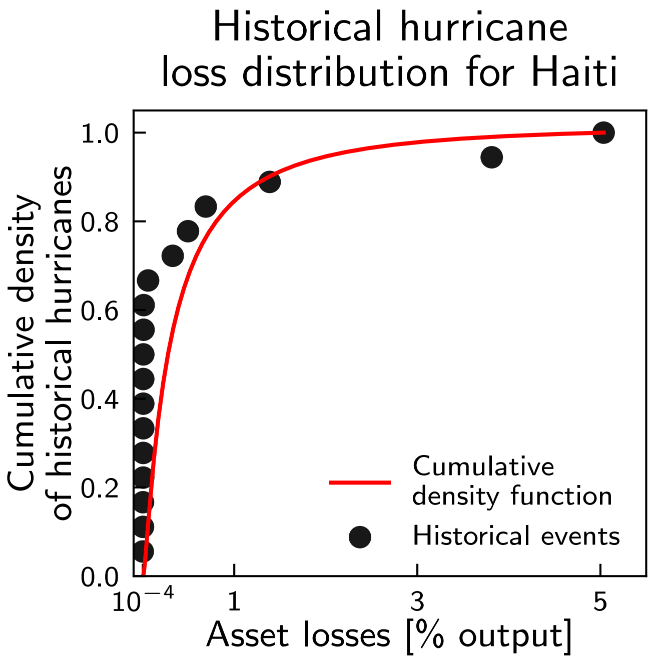

Fig. S15. Distribution of the historical asset losses of hurricanes that made landfall in Haiti in the period 1980–2014.
Black dots depict the asset losses as reported by the NatCatSERVICE database *(1)* in the period 1980–2014 relative to Haiti's growth domestic product of the years in which the hurricanes made landfall. The red line depicts a fit with a log-normal cumulative density function.

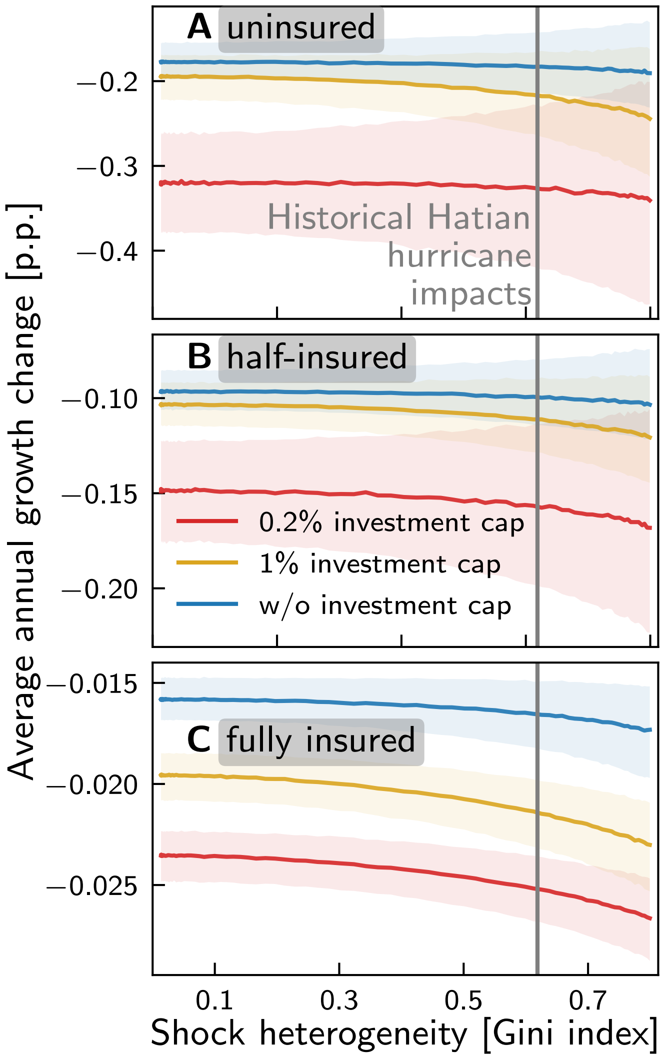

Fig. S16. Impact of hurricane shock heterogeneity on annual output growth rate of Haiti. Median annual growth rate change of the Haitian economy under hurricane shocks relative to the growth rate of the corresponding unperturbed economy, as a function of shock heterogeneity – measured by the Gini index – for no (**A**), half (**B**), and full (**C**) insurance coverage. Blue, ochre, and red lines depict median growth rate changes for scenarios where reconstruction investment is not limited, limited to 0.2%, and 1% of weekly output, respectively; shaded areas mark the corresponding 16.7-83.3 percentile confidence intervals. The grey vertical line indicates the median Gini index of the historical distribution of relative asset losses. In each simulation run, the Haitian GDP per capita (1402.1 USD) grows initially with 1.95% per year and is threatened by 18 hurricanes with landfall within 33 years, which add up to 11.86% capital damage.

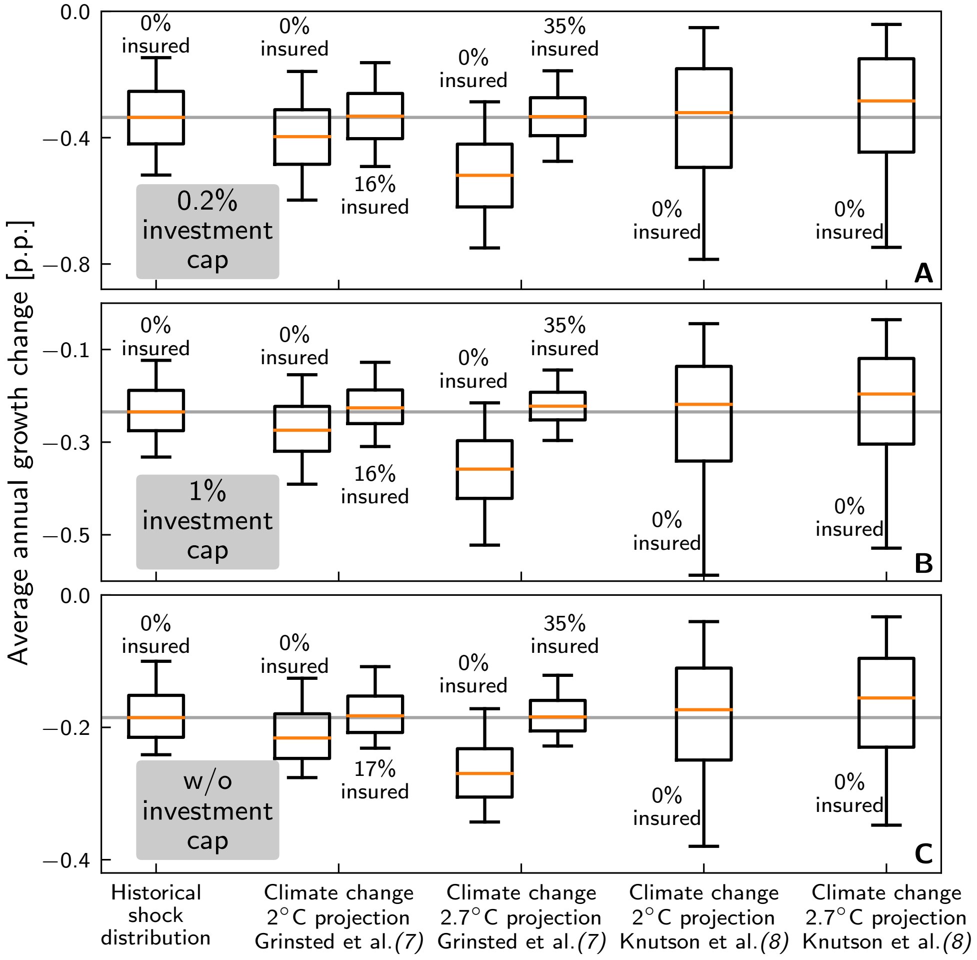

Fig. S17. Projected impacts of hurricanes on economic growth in 2°C and 2.7°C worlds and the effectiveness of insurance as coping strategy for Haiti. Annual growth losses (relative to the corresponding unperturbed economies evolving on the balanced growth paths) as obtained for the historical shock distribution (0% insurance coverage, period 1980-2012; 1st column), for Paris-compatible +2°C warming above pre-industrial levels (2nd, 3rd, 6th and 7th column) and +2.7°C (4th, 5th, 8th and 9th column) warming in compliance with current policies for reconstruction investment caps of 0.2% (**A**, standard scenario), 1% (**B**) and without reconstruction investment cap (**C**). Climate change projections of growth losses are derived from two different methods to estimate climate change-induced changes in hurricane climatology by Grinsted et al. *(7)* and Knutson et al. *(8)* (0% insurance coverage, 2nd and 4th column, respectively). Additionally, for both estimates and warming levels, the insurance coverages that would be necessary to reduce growth losses to the historical level are shown (3rd, 5th, 7th, and 9th column). Orange lines, boxes, and whiskers indicate median loss estimates as well as the 25th-75th and 5th-95th percentile ranges, respectively.

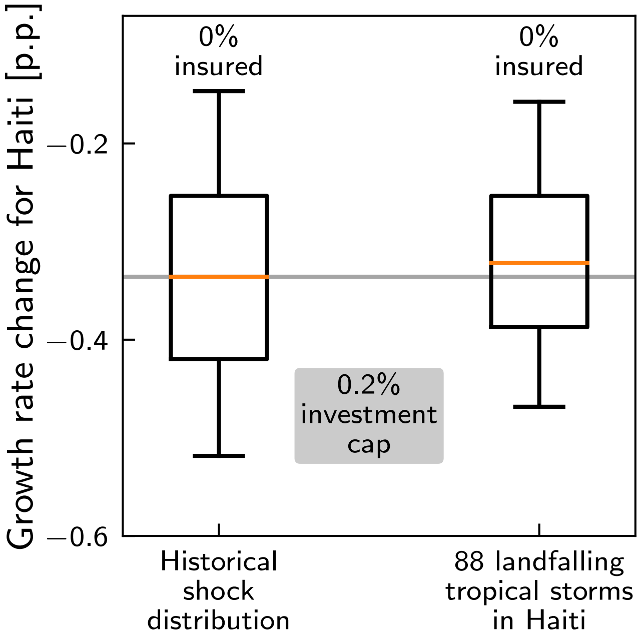

Fig. S18. Haitian growth losses in dependence of hurricane number. Average annual growth losses in Haiti (relative to the corresponding unperturbed baseline scenario) as obtained for the cumulative relative asset losses of 11.86% as reported in the historical period and the median historical shock heterogeneity (Gini index of 0.58) as obtained for 18 storms (number of reported storm in Haiti over the period 1980–2014, left) and 88 storms (number of reported storms in the US over the period 1980–2014, right). Orange lines, boxes, and whiskers indicate median loss estimates as well as the 25th-75th and 5th-95th percentile ranges, respectively. Parameters: zero insurance coverage and reconstruction investment capped to 0.2% of weekly output.
